# The assembly of RAB22A/TMEM33/RTN4 initiates a secretory ER-phagy pathway

**DOI:** 10.1038/s41421-025-00792-2

**Published:** 2025-04-29

**Authors:** Xueping Zheng, Dongmei Fang, Hao Shan, Beibei Xiao, Denghui Wei, Yingyi Ouyang, Lanqing Huo, Zhonghan Zhang, Yuanzhong Wu, Ruhua Zhang, Tiebang Kang, Ying Gao

**Affiliations:** Sun Yat-sen University Cancer Center, State Key Laboratory of Oncology in South China, Guangdong Provincial Clinical Research Center for Cancer, Guangzhou, Guangdong China

**Keywords:** Macroautophagy, Autophagosomes, Endoplasmic reticulum

## Abstract

Rafeesome, a newly identified multivesicular body (MVB)-like organelle, forms through the fusion of RAB22A-mediated ER-derived noncanonical autophagosomes with RAB22A-positive early endosomes. However, the mechanism underlying the formation of RAB22A-mediated noncanonical autophagosomes remains unclear. Herein, we report a secretory ER-phagy pathway in which the assembly of RAB22A/TMEM33/RTN4 induces the clustering of high-molecular-weight RTN4 oligomers, leading to ER membrane remodeling. This remodeling drives the biogenesis of ER-derived RTN4-positive noncanonical autophagosomes, which are ultimately secreted as TMEM33-marked RAB22A-induced extracellular vesicles (R-EVs) via Rafeesome. Specifically, RAB22A interacts with the tubular ER membrane protein TMEM33, which binds to the TM2 domain of the ER-shaping protein RTN4, promoting RTN4 homo-oligomerization and thereby generating RTN4-enriched microdomains. Consequently, the RTN4 microdomains may induce high curvature of the ER, facilitating the bud scission of RTN4-positive vesicles. These vesicles are transported by ATG9A and develop into isolation membranes (IMs), which are then anchored by LC3-II, a process catalyzed by the ATG12-ATG5-ATG16L1 complex, allowing them to grow into sealed RTN4 noncanonical autophagosome. While being packaged into these ER-derived intermediate compartments, ER cargoes bypass lysosomal degradation and are directed to secretory autophagy via the Rafeesome-R-EV route. Our findings reveal a secretory ER-phagy pathway initiated by the assembly of RAB22A/TMEM33/RTN4, providing new insights into the connection between ER-phagy and extracellular vesicles.

## Introduction

Autophagy is a highly conserved catabolic process that is essential for cellular quality control, as it facilitates the sequestration and/or degradation of excess cytosolic components and damaged organelles^1,2^. The dynamic formation of autophagosomes is tightly linked to the reorganization of the membrane system, which is orchestrated by autophagy-related genes (ATGs)^3,4^. Briefly, isolation membranes (IMs, also called phagophores) arise at the phagophore assembly site (PAS), to which the ATG12-ATG5-ATG16L1 complex is recruited to catalyze the conversion of LC3-I to lipidated LC3 (LC3-II), a well-recognized marker for autophagosomes, thereby facilitating the expansion of phagophores into double-membrane autophagosomes^5–9^. Rafeesomes, recently identified MVB-like structures, are formed by the fusion of RAB22A-mediated ER-derived noncanonical autophagosomes with RAB22A-positive early endosomes^10^. Similar to multivesicular bodies (MVBs), Rafeesomes fuse with the plasma membrane to release inner noncanonical autophagosomes. Ge et al. categorized this pathway into secretory autophagy, a key trafficking route that mediates the secretion of various substrates relying on non-degradative autophagosomes^11^. However, the source of IMs for RAB22A-mediated ER-derived secretory autophagosomes remains largely unknown.

The endoplasmic reticulum (ER), a continuous membranous organelle widely distributed in cells, comprises the nuclear envelope (NE) and the peripheral ER^12,13^. The peripheral ER forms an extensive interconnected network consisting of the sheet ER and the tubular ER^14^. Morphologically, the sheet ER is characterized by a flat cisternae-like structure with low membrane curvature, while the tubular ER is highly curved in cross-section^15,16^. The tubular ER network is maintained through a series of membrane-shaping protein families. Atlastins (ATLs), dynamin-like GTPases, mediate the homo-fusion of ER tubules to form three-way junctions^17,18^. Moreover, ER curvature is maintained by the Reticulon (RTN) and DP1/Yop1 families, which are exclusively concentrated at tubular ER and the edges of sheet ER^19–21^. In mammals, the Reticulon family contains four members (RTN1–4), among of which the RTN4 subfamily is the most studied protein responsible for inducing and stabilizing ER tubulation^15,22–25^. The RTN4 subfamily, including RTN4A, RTN4B and RTN4C, share a conserved RTN homology domain (RHD) comprising two highly hydrophobic short hairpin transmembrane (TM) domains connected by a cytoplasmic linker^19,26,27^. Due to the short hairpin topology, the RHD forms a “wedge-like” structure, occupying more space in the cytoplasmic leaflet than in the luminal leaflet^19,26^. This unique structural feature likely enables RTNs to generate and maintain the curvature of ER tubules. Indeed, RTNs tend to form immobile high-order oligomers, which in turn form arch-shaped clusters on the membrane to sustain the curved topology, thereby constricting and partitioning to ER tubules^21,26^. Electron microscopy (EM) studies have shown that ER sheets appear to be rough and densely studded with ribosomes, making them preferential for the synthesis and translation of proteins^28–30^. Conversely, the tubular ER tends to be smooth and is likely the primary regions for lipid synthesis and transport^30,31^. Although there is mounting evidence supporting the ER as the origin of autophagosomal membranes, the specific ER subdomain contributing to phagophore formation and how ER morphology associates with autophagy machinery still remains to be determined.

ER remodeling, including rearrangement, budding, membrane fusion and fragmentation, is pivotal to ER quality control and maintenance of cellular homeostasis in response to stress, as well as under normal conditions^32–34^. These processes frequently depend on the selective autophagic degradation machinery coined as macro-ER-phagy (commonly termed as ER-phagy)^35,36^. In general, ER-phagy occurs with the assistance of ER-phagy receptors including FAM134B, RTN3, ATL3 and CCPG1, which typically undergo oligomerization and bridge ER cargoes targeted for degradation with phagophore^37–40^. These receptors harbor an RHD domain that drives ER membrane bending and remodeling, which is critical to the occurrence of ER-phagy^19,32^. In addition to macro-ER-phagy, there are also micro-ER-phagy and ER-derived vesicle-mediated ER-phagy, both of which depend on lysosomal degradation of ER fragments, but are independent of canonical autophagy machinery^41,42^.

In this study, we identified a secretory ER-phagy pathway that mediates the biogenesis of RAB22A-mediated secretory autophagosomes, which we named RTN4 noncanonical autophagosomes. In this process, ER remodeling induced by the assembly of RAB22A/TMEM33/RTN4 results in the emergence of ER-derived RTN4 vesicles. Furthermore, we found that ATG9A is responsible for the trafficking of RTN4 vesicles for homo-fusion, providing precursors for RTN4 noncanonical autophagosomes. Our findings propose a novel secretory ER-phagy pathway and reveal that RTN4 noncanonical autophagosomes act as intermediate station for ER cargoes destined for secretion via Rafeesome-R-EV route.

## Results

### Both TMEM33 and RTN4B are enriched in Rafeesomes

**Fig. 1:**
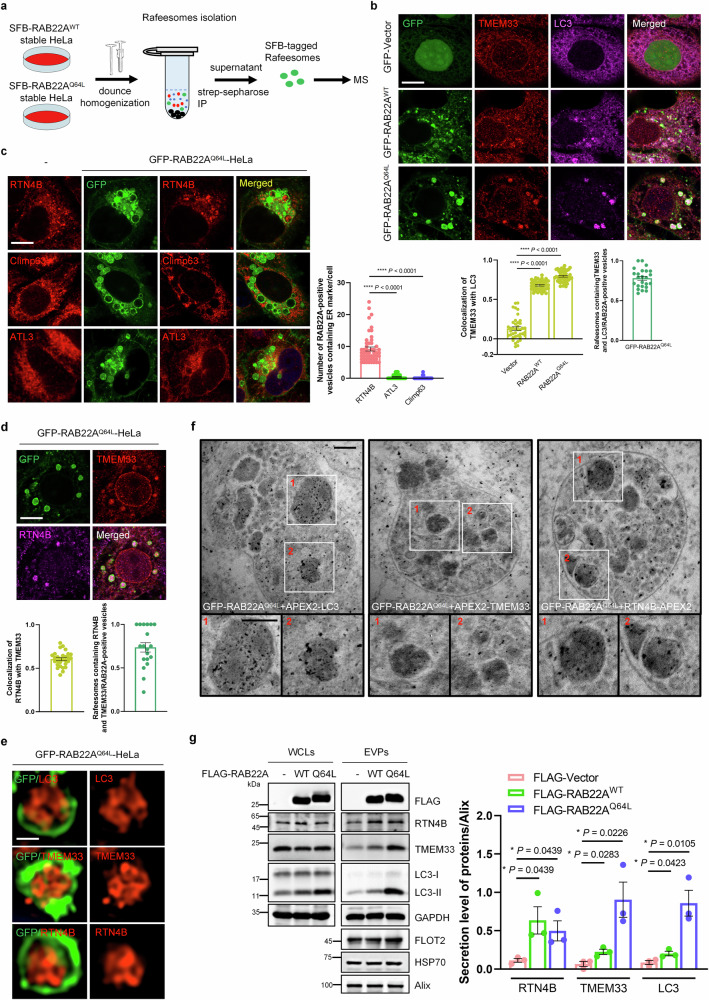
The ER membrane proteins TMEM33 and RTN4B are both enriched in Rafeesome. **a** Schematic diagram of the purification of Rafeesomes in HeLa cells stably expressing SFB-RAB22A^WT^ or SFB-RAB22A^Q64L^ using streptavidin-Sepharose followed by qualitative mass spectrometry analysis. **b** The colocalization of endogenous TMEM33 (red) and endogenous LC3 (magenta) with GFP-Vector, GFP-RAB22A^WT^ or GFP-RAB22A^Q64L^ was detected by immunofluorescence. Scale bar, 10 μm. Quantification of TMEM33 colocalization with LC3 was presented as Pearson’s correlation coefficient (*r*). The data are presented as means ± SEMs. *n* = 40, 41, 47 cells from three independent experiments. *p* values were calculated by Student’s *t*-test. ****p < 0.0001. The ratio of Rafeesomes induced by GFP-RAB22A^Q64L^ containing endogenous TMEM33 and LC3 was quantified and calculated relative to total RAB22A-positive vesicles. **c** The enrichment of the tubular ER markers RTN4B, ATL3 or the sheet ER marker Climp63 in Rafeesomes was examined using the indicated endogenous antibodies in HeLa cells stably expressing GFP-RAB22A^Q64L^. The left lane shows the distribution of the indicated proteins under steady-state conditions in WT HeLa cells. Scale bar, 10 μm. The data are presented as means ± SEMs. *n* = 51, 50, 52 cells from three independent experiments. *p* values were calculated by Student’s *t*-test. ***p < 0.0001. The number of RAB22A-positive vesicles containing corresponding ER marker was counted. **d** The colocalization of endogenous TMEM33 (red) with RTN4B (magenta) within Rafeesomes was assessed in HeLa cells stably expressing GFP-RAB22A^Q64L^. Scale bar, 10 μm. Quantification of TMEM33 colocalization with RTN4B was presented as Pearson’s correlation coefficient (*r*). *n* = 29 cells from three independent experiments. The ratio of Rafeesomes containing endogenous TMEM33 and RTN4B was quantified and calculated relative to total RAB22A-positive vesicles. **e** The distribution patterns of endogenous LC3, TMEM33 and RTN4B within Rafeesomes were determined using Structure Illumination Microscopy (SIM) imaging. Scale bar, 1 μm. **f** Electron microscopy (EM) of the APEX2-labeled LC3, TMEM33 and RTN4B in HeLa cells stably expressing GFP-RAB22A^Q64L^. Scale bar, 200 nm. **g** Extracellular vesicle particles (EVPs) derived from HeLa cells stably expressing vector, FLAG-RAB22A^WT^ or FLAG-RAB22A^Q64L^ were isolated using differential ultracentrifugation, and whole cell lysates (WCLs) and EVPs were subjected to western blotting using the indicated antibodies. The data are presented as means ± SEMs. *n* = 3. *p* values were calculated by Student’s *t*-test. *p < 0.05.

To identify the key molecules involved in the formation of RAB22A-mediated noncanonical autophagosomes, a sub-organelle isolation method was employed to precipitate Rafeesomes in HeLa cells stably expressing SFB-RAB22A^WT^ or SFB-RAB22A^Q64L^, the sub-organelles were subsequently analyzed by mass spectrometry (MS) (Fig. 1a). As expected, the precipitated Rafeesomes from HeLa cells stably expressing GFP-RAB22A^Q64L^, which had a diameter of approximately 1 – 5-μm, were intact (Supplementary Fig. S1a). Calnexin and LC3-II, two known protein cargoes of Rafeesomes, were clearly detected (Supplementary Fig. S1b). Given that RAB22A-mediated noncanonical autophagosomes are ER-derived^10^, we focused on ER membrane proteins with relatively high abundance identified by mass spectrometry (MS) from these cells (Supplementary Tables S1 and S2). As shown in Supplementary Fig. S1c, among these candidates, HA-TMEM33 colocalized with LC3-II and was enriched in Rafeesomes in HeLa cells stably expressing GFP-RAB22A^Q64L^. Moreover, the colocalization of endogenous TMEM33 with GFP-RAB22A^WT^ or GFP-RAB22A^Q64L^ was observed (Fig. 1b). Similarly, HA-TMEM33 also colocalized with endogenous RAB22A (Supplementary Fig. S1d). These results suggested that TMEM33 may be involved in the RAB22A-mediated ER-derived noncanonical autophagy.

Given that TMEM33 specifically resides in the tubular ER, we hypothesized that the autophagosomal membranes required for RAB22A-mediated noncanonical autophagy may originate from the tubular ER. To substantiate this, two tubular ER markers, RTN4 (i.e., the RTN4B isoform) and ATL3, as well as the sheet ER marker Climp63 were used. RTN4B, but not ATL3 or Climp63, strongly colocalized with LC3-II within Rafeesomes (Fig. 1c; Supplementary Fig. S1e), and the colocalization of endogenous RTN4B with RAB22A was also detected (Supplementary Fig. S1f), indicating that RTN4B-resident tubular ER provides membrane components for RAB22A-mediated noncanonical autophagy. Moreover, the endogenous TMEM33 and RTN4B were co-enriched in Rafeesomes (Fig. 1d). Using super-resolution structured illumination microscopy (SIM), similar to LC3-II, either endogenous or exogenous TMEM33 or RTN4B was observed in intraluminal vesicle (ILV)-like structures with diameters ranging from 0.2 to 0.5 μm within Rafeesomes (Fig. 1e; Supplementary Fig. S1g). To observe LC3-, TMEM33- or RTN4B-localized structures within Rafeesomes, we performed APEX2-DAB staining followed by EM analysis. In Fig. 1f, we observed APEX2-positive single-membrane vesicular bodies within Rafeesome in HeLa cells stably expressing GFP-RAB22A^Q64L^. In addition, HeLa cells stably expressing FLAG-RAB22A^WT^ or FLAG-RAB22A^Q64L^ promotes the secretion of TMEM33 and RTN4B into R-EVs (Fig. 1g). Based on the fact that RTNs form arch-shaped clusters on the membrane to maintain a curved topology^19^, these results suggested that RTN4 also likely contributes to the formation of RAB22A-mediated ER-derived noncanonical autophagosomes by inducing ER remodeling.

### TMEM33 and RTN4 are required for RAB22A-mediated noncanonical autophagy

**Fig. 2:**
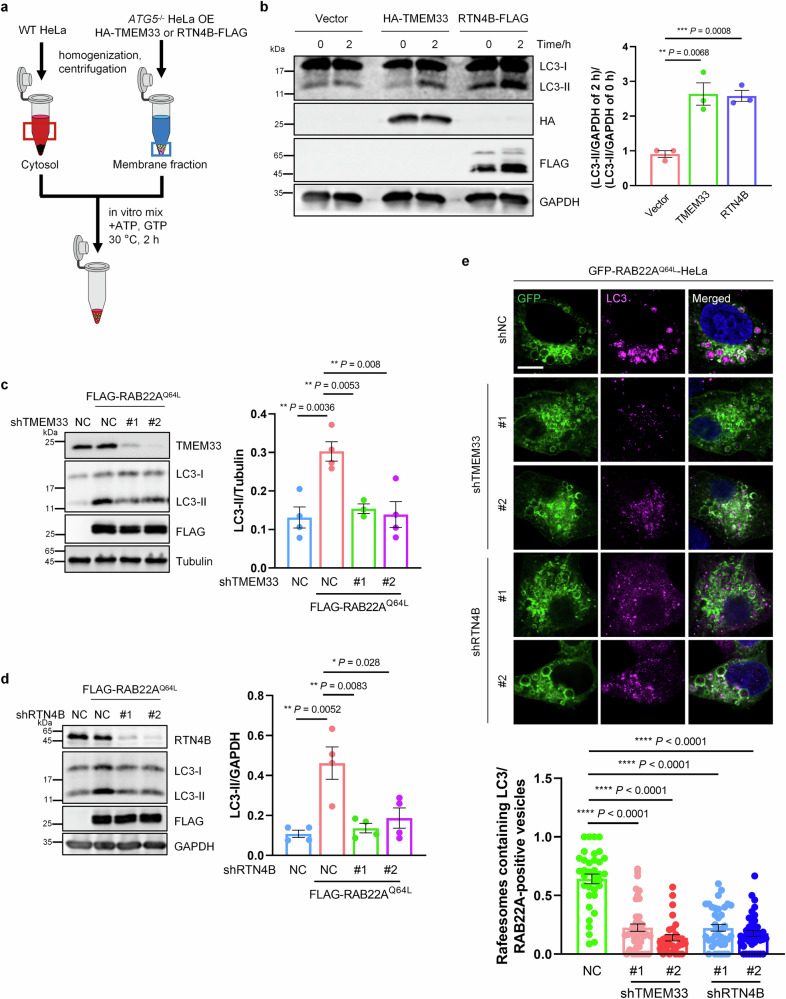
TMEM33 and RTN4 are required for RAB22A-mediated noncanonical autophagy in the ER. **a** Diagram showing the workflow for the in vitro reconstitution of LC3 lipidation. **b** The mixture containing the cytosol of WT HeLa cells and the membrane fractions of *ATG5*^*–/–*^ HeLa-HA-TMEM33 or -RTN4B-FLAG cells was analyzed using western blotting, and the levels of lipidated LC3 (LC3-II) were measured. The data are presented as means ± SEMs. *n* = 3. *p* values were calculated by Student’s *t*-test. **p < 0.01, ***p < 0.001. **c**, **d** LC3-II levels in HeLa cells with stable FLAG-RAB22A^Q64L^ expression and TMEM33 (**c**) or RTN4B (**d**) knockdown were analyzed by Western blotting. The data are presented as means ± SEMs. *n* ≥ 3. *p* values were calculated by Student’s *t*-test. *p < 0.05, **p < 0.01. **e** Immunofluorescence of LC3 in HeLa cells stably expressing GFP-RAB22A^Q64L^ with TMEM33 or RTN4B knockdown. The Ratio of Rafeesomes with a diameter of 1–5 μm and containing endogenous LC3 was calculated relative to total RAB22A-positive vesicles. Scale bar, 10 μm. The data are presented as means ± SEMs. *n* ≥ 30 cells from three independent experime*n*ts. *p* values were calculated by Student’s *t*-test. ****p < 0.0001.

To explore the potential roles of both TMEM33 and RTN4 in RAB22A-mediated noncanonical autophagy, we first examined whether TMEM33 or RTN4 is involved in autophagy. Indeed, the overexpression of TMEM33 or RTN4B increased the LC3-II level (Supplementary Fig. S2a, b), whereas the knockdown of TMEM33 or RTN4B decreased the basal LC3-II level (Supplementary Fig. S2c, d), indicating that both TMEM33 and RTN4B may trigger autophagy. For further substantiation, a well-established in vitro LC3 lipidation system was performed, in which cytosol from WT HeLa cells and membrane fractions from *ATG5*^–/–^ HeLa cells transiently expressing vector, HA-TMEM33 or RTN4B-FLAG were incubated in the presence of an ATP regeneration system (Fig. 2a). Expectedly, LC3 conversion clearly increased with TMEM33 or RTN4B expression (Fig. 2b; Supplementary Fig. S2e, f). Importantly, the downregulation of TMEM33 or RTN4B impaired the induction of RAB22A^Q64L^-mediated noncanonical autophagy and the formation of Rafeesomes (Fig. 2c–e), as well as the secretion of LC3-II into R-EVs (Supplementary Fig. S2g, h). Collectively, these results revealed that RAB22A-mediated ER-derived secretory autophagy is dependent on TMEM33 and RTN4.

### TMEM33 acts as a marker for RAB22A-mediated secretory autophagy

**Fig. 3:**
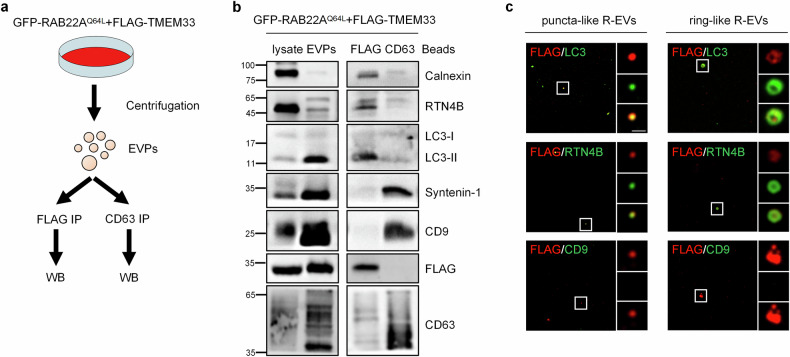
TMEM33-marked R-EVs are distinct from classical exosomes. **a** Schematic diagram depicting the workflow for the immunoaffinity approach for isolating FLAG-tagged R-EVs and CD63-positive classical exosomes from total EVPs collected from HeLa cells stably expressing both GFP-RAB22A^Q64L^ and FLAG-TMEM33. **b** The cell lysates, total EVPs and anti-FLAG or anti-CD63 bead-conjugated EV populations were analyzed via western blotting using the indicated antibodies. **c** The purified FLAG-TMEM33-labeled R-EVs eluted with FLAG peptides were attached to coverslips precoated with 0.2 mg/mL poly-L-lysine. Then the R-EVs were stained with the indicated antibodies. Scale bar, 1 μm.

Given that TMEM33 can be transported via the RAB22A-mediated Rafeesome-R-EV route and TMEM33 is a key molecule in RAB22A-mediated noncanonical autophagy, we assumed that TMEM33 could be used as a marker of RAB22A-mediated secretory autophagy. To confirm this, as illustrated in Fig. 3a, an immunoaffinity approach was designed in which total EVPs collected from cells simultaneously expressing GFP-RAB22A^Q64L^ and FLAG-TMEM33 were equally divided into two aliquots, followed by incubation with either anti-FLAG beads or anti-CD63 beads. As predicted, calnexin, RTN4B and LC3-II were enriched in FLAG-TMEM33-labeled R-EVs but not in CD63-positive exosomes (Fig. 3b). In contrast, Sytenin-1 and CD9, both known to be present in classical exosomes, were absolutely absent from TMEM33-labeled R-EVs (Fig. 3b). To visualize R-EVs, the purified FLAG-TMEM33-labeled R-EVs eluted with FLAG peptides were seeded onto coverslips precoated with poly-L-lysine, and the localization of RTN4B and LC3-II, but not CD9, on R-EVs was clearly observed. Notably, most of the FLAG-TMEM33-labeled R-EVs exhibited puncta-like structures approximately 0.2 μm in diameter, while few of them displayed ring-like vesicles with diameters of about 0.5 μm (Fig. 3c). The sizes of FLAG-TMEM33-labeled R-EVs were similar to those of RTN4 noncanonical autophagosomes, but different from those of classical exosomes (50 – 150 nm)^43^. These results indicated that TMEM33-positive R-EVs represent a specific class of EV group which contains ER-derived cargoes, establishing TMEM33 as a marker for RAB22A-mediated secretory autophagy.

### RAB22A and TMEM33 promote RTN4 oligomerization to form RTN4 puncta

**Fig. 4:**
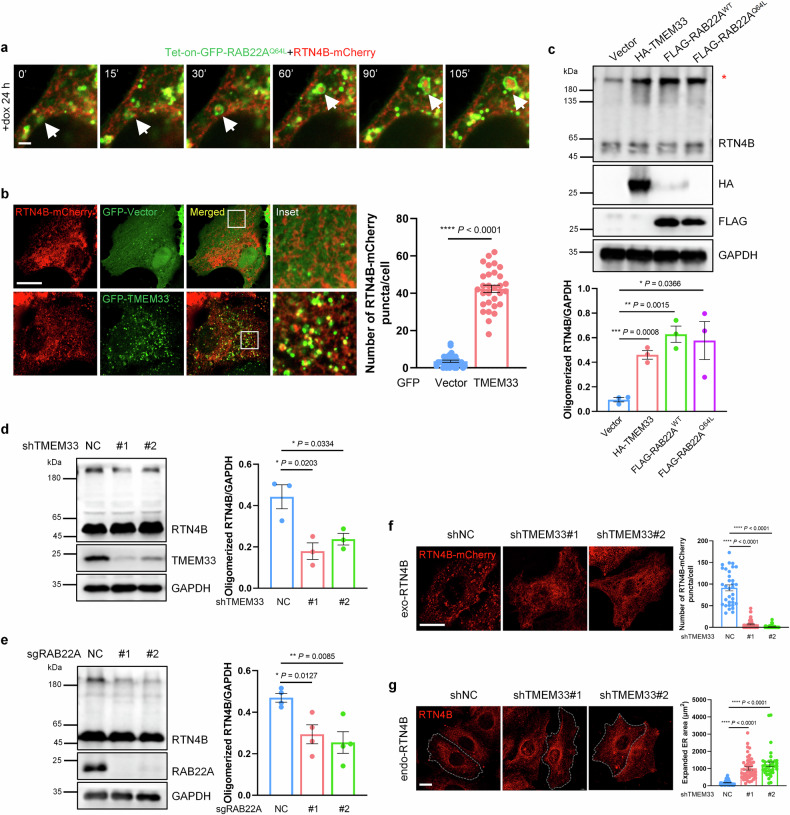
RAB22A and TMEM33 facilitate RTN4 oligomerization to form RTN4 puncta. **a** Tet-on-GFP-RAB22A^Q64L^ stable HeLa cells were transiently transfected with RTN4B-mCherry for 24 h, followed by the treatment with 50 ng/mL dox for 24 h. Then time-lapse imaging was performed with a Nikon Ti2 spinning disk living cell microscope. RTN4B-mCherry puncta were formed and entered into Rafeesomes. Scale bar, 2 μm. **b** Immunofluorescence of HeLa cells transiently co-transfected with RTN4B-mCherry (red) and GFP-Vector or GFP-TMEM33 for 24 h. The number of RTN4B-mCherry puncta was quantified. Scale bar, 10 μm. The data are presented as means ± SEMs. *n* = 31, 30 cells from three independent experiments. *p* values were calculated by Student’s *t*-test. ****p < 0.0001. **c** HeLa cells transiently transfected with HA-TMEM33, FLAG-RAB22A^WT^ or FLAG-RAB22A^Q64L^ were treated with 2 mM DSS, then the cell lysates were subjected to western blotting to assess endogenous RTN4B oligomerization indicated by a red asterisk. The data are presented as means ± SEMs. *n* = 3. *p* values were calculated by Student’s *t*-test. *p < 0.05, **p < 0.01, ***p < 0.001. Endogenous RTN4B oligomerization was assessed in HeLa cells with TMEM33 (**d**) or RAB22A (**e**) knockdown by western blotting. The data are presented as means ± SEMs. *n* ≥ 3. *p* values were calculated by Student’s *t*-test. *p < 0.05, **p < 0.01. **f** HeLa cells were transfected with RTN4B-mCherry with or without TMEM33 knockdown. RTN4B-mCherry puncta were counted. Scale bar, 10 μm. The data are presented as means ± SEMs. *n* = 33, 50, 45 cells from three independent experiments. *p* values were calculated by Student’s *t*-test. ****p < 0.0001. **g** ER area was measured in TMEM33-knockdown HeLa cells. Scale bar, 10 μm. The data are presented as means ± SEMs. *n* = 60, 48, 37 cells from three independent experiments. *p* values were calculated by Student’s *t*-test. ****p < 0.0001.

Next, we investigated the relationship between TMEM33 and RTN4 during RAB22A-mediated noncanonical autophagy. In fact, RAB22A^Q64L^ induced the formation of large RTN4B puncta within Rafeesomes (Fig. 1c), prompting us to primarily focus on these RTN4B puncta. Using a doxycycline (dox)-inducible expression system, we carried out real-time fluorescence imaging to observe RTN4B puncta formation. As shown in Supplementary Fig. S3a, an increase in the number of RTN4B-mCherry puncta was observed after the induction of GFP-RAB22A^Q64L^, indicating that RAB22A facilitated the formation of RTN4B puncta. Interestingly, RTN4B-mCherry puncta were engulfed by RAB22A^Q64L^-positive early endosomes to form Rafeesomes (Fig. 4a; Supplementary Fig. S3b), which may fuse with other Rafeesomes to form a large-sized Rafeesome (Fig. 4a). Similarly, GFP-TMEM33 also promoted the formation of RTN4B-mCherry puncta, which colocalized with TMEM33 (Fig. 4b). Considering that RTNs are able to form immobile oligomers on the tubular ER membrane^21^, we speculated that RTN4B puncta may result from RTN4B oligomerization. Indeed, in a dose-dependent manner, RTN4B formed higher-order complexes (approximately 180 kDa) in both HeLa and HEK293T cells, after treatment with disuccinimidyl suberate (DSS), a membrane-permeable cross-linker that reacts with primary amino groups (-NH2) to form stable amide bonds (Supplementary Fig. S3c). Furthermore, HA-TMEM33, FLAG-RAB22A^WT^ or FLAG-RAB22A^Q64L^ strongly enhanced RTN4 oligomerization (Fig. 4c), whereas the knockdown of either TMEM33 or RAB22A led to the decrease of endogenous RTN4B oligomerization (Fig. 4d, e). Moreover, TMEM33 knockdown reduced both RTN4B-FLAG oligomerization and RTN4B-mCherry puncta (Fig. 4f; Supplementary Fig. S3d), and simultaneously led to ER expansion, as indicated by an increase in Climp63-positive membranes (Fig. 4g; Supplementary Fig. S3e). These results suggested that excessive TMEM33 and RAB22A synergistically promote the formation of RTN4 cluster-enriched microdomains to form RTN4 puncta.

### The TM2 domain within the RHD of RTN4 is required for its oligomerization and interaction with TMEM33

**Fig. 5:**
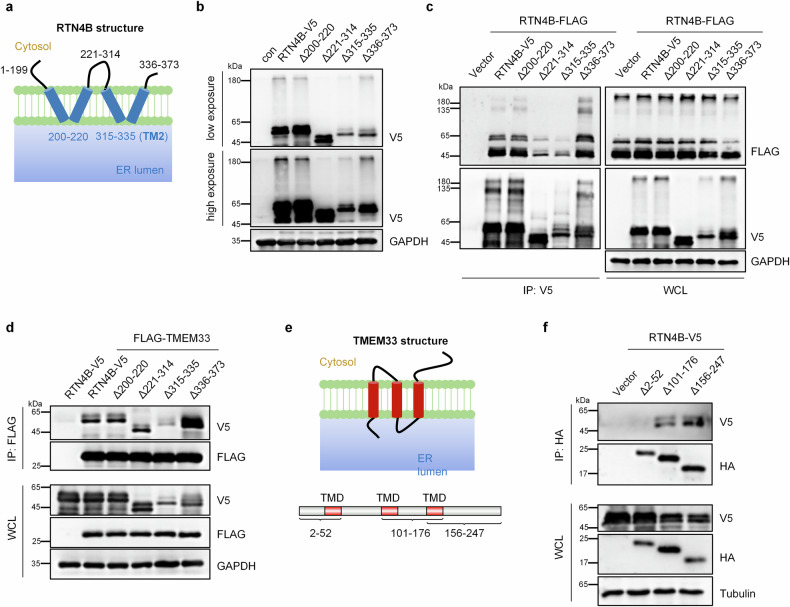
The TM2 domain of RTN4 is required for its oligomerization and interaction with TMEM33. **a** A sketch map of the RTN4B transmembrane topology structure in the ER membrane. **b** HeLa cells were transiently transfected with RTN4B-truncation mutants according to **a**, and RTN4B oligomerization was assessed via western blotting. **c** HEK293T cells were co-transfected with FLAG-tagged RTN4B and V5-tagged RTN4B-truncation mutants, then the cell lysates were incubated with V5 agarose for 6 h at 4 °C, after which western blotting was performed to assess their interaction. **d** HEK293T cells were co-transfected with FLAG-TMEM33 and RTN4B-truncation mutants, and immunoprecipitation was subsequently performed using FLAG agarose. **e** Schematic representation of the TMEM33 transmembrane topology structure in the ER membrane. **f** Cell lysates extracted from HEK293T cells co-transfected with RTN4B-V5 and the indicated HA-tagged TMEM33-truncation mutants were incubated with HA agarose and were then subjected to western blotting.

To determine how TMEM33 induces RTN4 oligomerization in the ER, we constructed truncated RTN4B mutants based on its topology structure (Fig. 5a). Deletion of amino acids (aa) 221 – 314 or the second transmembrane domain (TM2, aa 315–335) within the RHD markedly decreased the amount of oligomeric RTN4B (Fig. 5b), which primarily existed as homo-oligomers (Fig. 5c), indicating that aa 221– 335 was primarily responsible for RTN4B homo-oligomerization. The TM2 domain of RTN4B was also found to be essential for its interaction with TMEM33 (Fig. 5d). Furthermore, the first transmembrane domain of TMEM33 (aa 2 – 52) was required for its interaction with RTN4B (Fig. 5e, f). These results indicated that TMEM33 binds to the TM2 domain of RTN4 to induce RTN4 homo-oligomerization, which likely leads to the morphological changes of the ER and promotes RTN4 budding to form RTN4 puncta.

The RTN4 subfamily contains three members, i.e., RTN4A, RTN4B and RTN4C, which share a highly conserved RHD domain containing the TM2 region of RTN4 (Supplementary Fig. S4a). We surmised that in addition to RTN4B, RTN4A and RTN4C may also bind to TMEM33 via their TM2 domain, thereby participating in RAB22A-mediated noncanonical autophagy in their oligomerized form. Indeed, RTN4A, RTN4C and the RHD domain of RTN4A alone (RTN4A^RHD^) could be packaged into Rafeesomes (Supplementary Fig. S4b). Additionally, RTN4C and RTN4A^RHD^ also formed immobile oligomers in a DSS dose-dependent manner (Supplementary Fig. S4c). Considering that RTN4B is the major RTN4 isoform expressed in non-neuronal cells^44^, we proposed that RTN4B is a major member of the RTN4 family that contributes to the formation of RAB22A-mediated noncanonical autophagosomes.

### RTN4 vesicles are precursors of RAB22A-mediated noncanonical autophagosomes

**Fig. 6:**
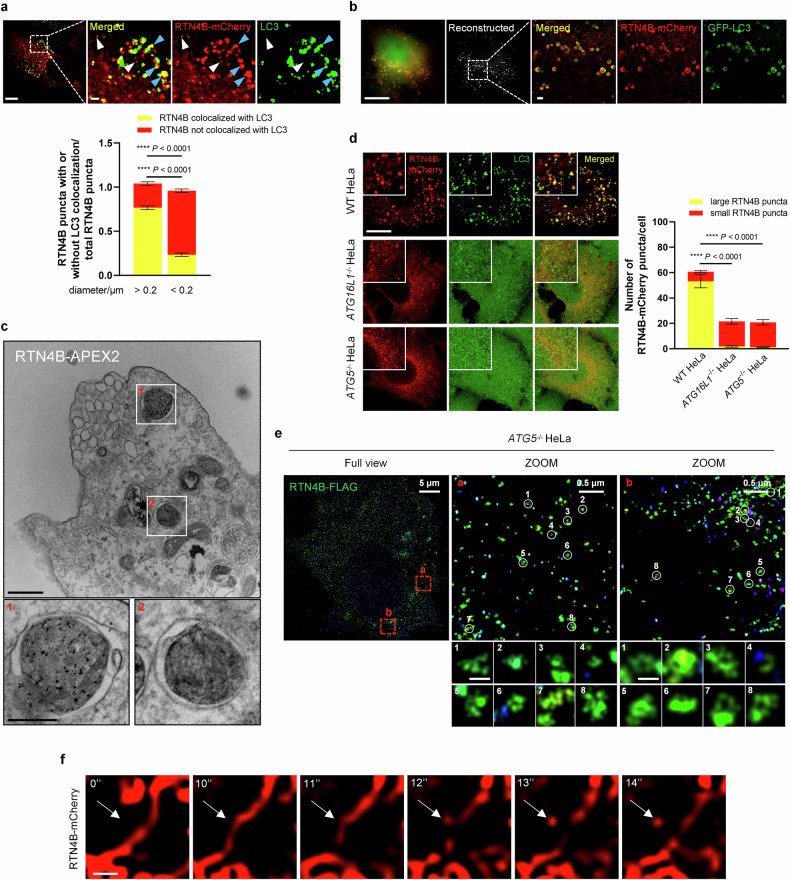
RTN4 vesicles are precursors of RAB22A-mediated noncanonical autophagosomes. **a** Immunofluorescence of RTN4B-mCherry (red) and endogenous LC3 (green) in HeLa cells transfected with RTN4B-mCherry for 36 h. The smaller RTN4B-mCherry puncta with diameters less than 0.2 μm are indicated by white arrows, and the larger RTN4B-mCherry puncta with diameters greater than 0.2 μm are denoted by blue arrows. Scale bar, 10 μm. The data are presented as means ± SEMs. *n* = 20 cells from three independent experiments. *p* values were calculated by Student’s *t*-test. ****p < 0.0001. **b** Multi-SIM analysis of large RTN4B-mCherry puncta in HeLa cells transiently co-transfected with RTN4B-mCherry and GFP-LC3 for 36 h. Scale bar, 10 μm. **c** EM analysis of APEX2-labeled RTN4B-positive structures in HeLa cells transfected with RTN4B-APEX2. Scale bar, 200 nm. **d** WT, *ATG16L1*^*–/–*^ and *ATG5*^*–/–*^ HeLa cells were individually transfected with RTN4B-mCherry for 36 h, after which the larger and smaller RTN4B-mCherry puncta with or without endogenous LC3 colocalization were quantified. Scale bar, 10 μm. The data are presented as means ± SEMs. *n* = 23, 22, 24 cells from three independent experiments. *p* values were calculated by Student’s *t*-test. ****p < 0.0001. **e** 3D-Stochastic optical reconstruction microscopy (STORM) analysis of small RTN4B-FLAG puncta in *ATG5*^*–/–*^ HeLa cells transiently transfected with RTN4B-FLAG. Scale bar, 100 nm. **f** Living cell imaging of the bud scission of RTN4B-mCherry puncta in HeLa cells transiently transfected with RTN4B-mCherry. Scale bar, 0.5 μm.

Next, we studied whether there was a correlation between ER-derived RTN4 puncta and RAB22A-mediated noncanonical autophagosomes. Interestingly, as shown in Fig. 6a, the larger RTN4B-mCherry puncta that substantially colocalized with LC3-II were about 0.2 – 0.5 μm in diameter, which was similar to the sizes of RAB22A-mediated noncanonical autophagosomes, while the smaller RTN4B-mCherry puncta with diameters less than 0.2 μm hardly colocalized with LC3-II. SIM analysis showed that the larger RTN4B-mCherry puncta exhibited vesicular structure with pronounced GFP-LC3 colocalization (Fig. 6b), and the EM analysis showed APEX2-positive double-membrane structures in HeLa cells expressing RTN4B-APEX2 (Fig. 6c). Notably, deletion of *ATG16L1* or *ATG5* significantly abolished the formation of larger RTN4B-mCherry puncta but had no effect on the smaller puncta (Fig. 6d; Supplementary Fig. S5a). By means of 3D-stochastic optical reconstruction microscopy (STORM), substantial vesicle-like structures in a diameter of 30 – 50 nm were observed in *ATG5*^*–/–*^ HeLa cells with transient overexpression of RTN4B-FLAG (Fig. 6e). Interestingly, these vesicles displayed a preference for clustering (Supplementary Fig. S5b). Meanwhile, using live cell imaging, we captured direct budding of RTN4B-mCherry puncta on the tubular ER (Fig. 6f; Supplementary Video S1). These observations suggested that RTN4 microdomains generate plenty of vesicular structures, which we hereafter called RTN4 vesicles, after budding off from the ER membrane. Considering that the formation of RAB22A-mediated noncanonical autophagosomes is dependent on the ATG12-ATG5-ATG16L1 complex^10^, we speculated that these RTN4 vesicles likely act as precursor membranes of the larger RTN4 puncta with LC3-II attachment through gathering. Hence, we named the larger RTN4 puncta “RTN4 noncanonical autophagosomes”, namely RAB22A-mediated secretory autophagosomes, which carry RAB22A, TMEM33, RTN4 and LC3-II. Oligomerized RTN4-induced non-degradative ER-phagy drove the formation of RTN4 noncanonical autophagosomes, which could be intermediate station for substrates of RAB22A-mediated Rafeesome-R-EV secretory pathway.

### TMEM33 promotes the formation of RTN4 noncanonical autophagosomes

**Fig. 7:**
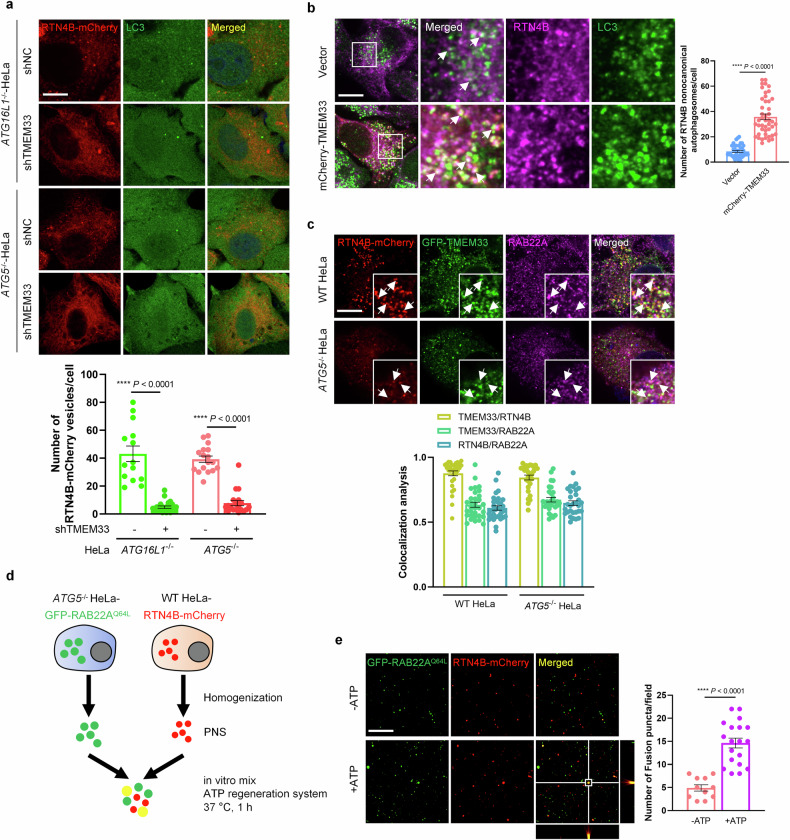
TMEM33 promotes the formation of RTN4 noncanonical autophagosomes. **a** *ATG16L1*^*–/–*^ and *ATG5*^*–/–*^ HeLa cells with or without TMEM33 knockdown were transfected with RTN4B-mCherry, after which the number of RTN4B-mCherry vesicles was counted. Scale bar, 10 μm. The data are presented as means ± SEMs. *n* > 15 cells from three independent experiments. *p* values were calculated by Student’s *t*-test. ****p < 0.0001. **b** HeLa cells were transfected with Vector or mCherry-TMEM33, and the number of endogenous RTN4B noncanonical autophagosomes colocalized with endogenous LC3 denoted by white arrows was quantified. Scale bar, 10 μm. The data are presented as means ± SEMs. *n* = 40, 45 cells from three independent experiments. *p* values were calculated by Student’s *t*-test. ****p < 0.0001. **c** Colocalization analysis of GFP-TMEM33 and endogenous RAB22A on RTN4B-mCherry noncanonical autophagosomes (upper lane) and RTN4B-mCherry vesicles (lower lane) in WT and *ATG5*^*–/–*^ HeLa cells co-transfected with RTN4B-mCherry and GFP-TMEM33. Scale bar, 10 μm. Quantification of colocalization was presented as Pearson’s correlation coefficient (*r*). *n* = 32, 30 cells from three independent experiments. **d** Working model for the in vitro fusion assay between GFP-labeled RAB22A^Q64L^ early endosomes and mCherry-labeled RTN4B noncanonical autophagosomes. **e** Fusion puncta (yellow), which indicate Rafeesomes, were quantified after captured using a super-resolution confocal microscopy. The represented merged puncta were shown on the *xy*, *xz* and *yz* axis. Scale bar, 10 μm. The data are presented as means ± SEMs. *n* = 11, 19 fields from three independent experiments. *p* values were calculated by Student’s *t*-test. ****p < 0.0001.

The above results showed that RTN4 formed high molecular weight oligomers via TMEM33 and RAB22A, thereby budding from membrane to form vesicular structures. Indeed, the knockdown of TMEM33 in *ATG16L1*^*–/–*^ or *ATG5*^*–/–*^ HeLa cells decreased the number of RTN4B-mCherry vesicles (Fig. 7a), and TMEM33 overexpression efficiently enhanced the formation of endogenous RTN4B noncanonical autophagosomes (Fig. 7b). These results are consistent with the finding that TMEM33 promotes RTN4 oligomerization to form RTN4 puncta. Furthermore, GFP-TMEM33 and endogenous RAB22A were readily detected on both mCherry-labeled RTN4B vesicles and RTN4B noncanonical autophagosomes (Fig. 7c). Notably, RTN4B^ΔTM2^ (Δ315 – 335), which failed to interact with TMEM33, lost the ability to form either RTN4B noncanonical autophagosomes or Rafeesomes (Supplementary Fig. S6a, b). Taken together, these findings revealed that the coordination of RAB22A with TMEM33 drives RTN4 budding to form RTN4 vesicles, which function as precursors for RAB22A-mediated noncanonical autophagosomes.

According to the definition of Rafeesome, RTN4 noncanonical autophagosomes should be able to fuse with RAB22A-positive early endosomes. For verification, a well-established in vitro membrane fusion assay was carried out (Fig. 7d). In this method, post-nuclear supernatants (PNSs) were extracted separately either from *ATG5*^*–/–*^ HeLa cells stably expressing GFP-RAB22A^Q64L^, in which RAB22A induces pure endosomes, or from WT HeLa cells stably expressing RTN4B-mCherry. Then, these two PNSs were incubated in vitro to monitor the membrane fusion events. As expected, yellow signals indicating the fusion between GFP-labeled RAB22A early endosomes with mCherry-labeled RTN4B noncanonical autophagosomes were clearly detected in the presence of ATP (Fig. 7e), and the Rafeesome structures were also observed (Supplementary Fig. S6c). These results confirmed that RTN4 noncanonical autophagosomes are capable of fusing with early endosomes to generate Rafeesomes. Previous studies have demonstrated that RAB22A inactivates RAB7, thereby preventing the fusion of RAB22A-positive autophagosomes with lysosomes^10^. This inhibition is likely mediated through the RAB22A-dependent recruitment of TBC1D2B, a GTPase-activating protein (GAP) for RAB7, as the presence of TBC1D2B in autophagosomes and its interaction with RAB22A were clearly detected (Supplementary Fig. S6d, e). The protection of RTN4 noncanonical autophagosomes from being degraded was further confirmed by using RTN4B with a tandem fusion of mCherry and GFP (RTN4B-mCherry-GFP), as indicated by that mCherry-positive puncta were almost colocalized with GFP-positive puncta (Supplementary Fig. S6f). As a consequence, ER-derived RTN4 noncanonical autophagosomes are secreted as R-EVs through Rafeesomes, escaping from lysosomal degradation.

Taken together, these data revealed that RAB22A/TMEM33/RTN4 assembly initiates a secretory ER-phagy pathway. This pathway drives the formation of RTN4 noncanonical autophagosomes, which package ER-derived substrates for secretion into extracellular space via the Rafeesome-R-EV route.

### ATG9A transports RTN4 vesicles to deliver membranes for RTN4 noncanonical autophagosomes

**Fig. 8:**
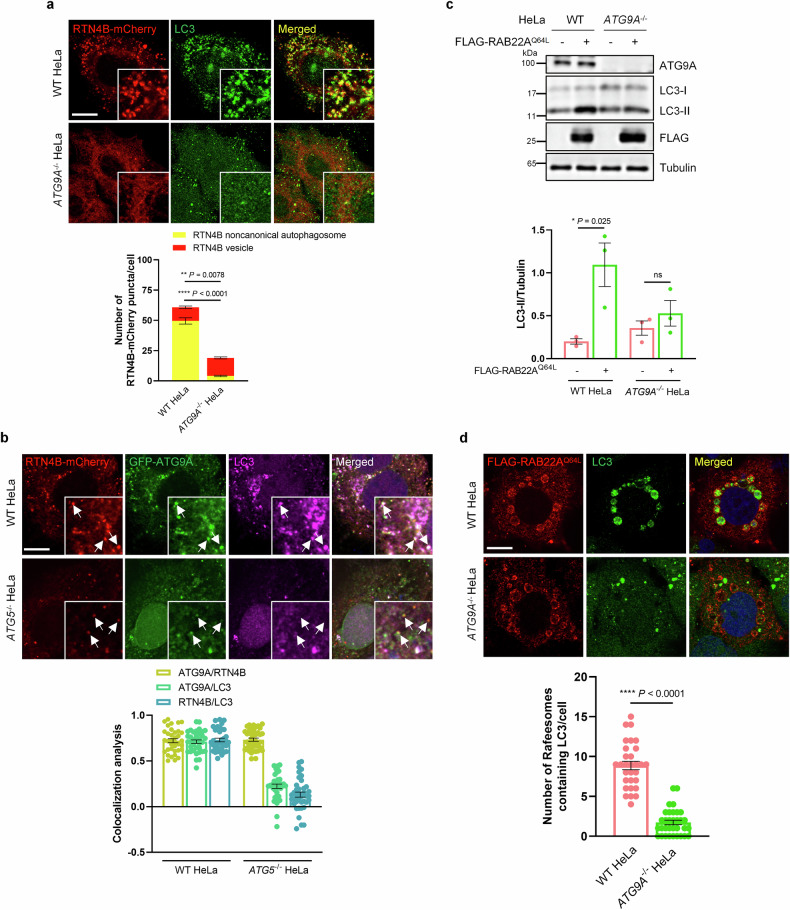
ATG9A transports RTN4 vesicles to promote the formation of RTN4 noncanonical autophagosomes. **a** WT or *ATG9A*^*–/–*^ HeLa cells were transfected with RTN4B-mCherry for 36 h, after which the formation of RTN4B-mCherry noncanonical autophagosomes colocalized with endogenous LC3 and RTN4B-mCherry vesicles were monitored. Scale bar, 10 μm. The data are presented as means ± SEMs. *n* = 22, 29 cells from three independent experiments. *p* values were calculated by Student’s *t*-test. **p < 0.01, ****p < 0.0001. **b** Colocalization analysis of GFP-ATG9A and endogenous LC3 on RTN4B-mCherry noncanonical autophagosomes (upper lane) or RTN4B-mCherry vesicles (lower lane) in WT or *ATG5*^*–/–*^ HeLa cells co-transfected with RTN4B-mCherry and GFP-ATG9A. Scale bar, 10 μm. Quantification of colocalization was presented as Pearson’s correlation coefficient (*r*). *n* = 36, 39 cells from three independent experiments. **c** LC3-II levels in WT and *ATG9A*^*–/–*^ HeLa cells with or without FLAG-RAB22A^Q64L^ overexpression were measured by western blotting. The data are presented as means ± SEMs. *n* = 3. *P* values were calculated by Student’s *t*-test. *p < 0.05, ns indicates not significant. **d** The formation of Rafeesomes containing endogenous LC3 was assessed in WT and *ATG9A*^*–/–*^ HeLa cells stably expressing FLAG-RAB22A^Q64L^. Scale bar, 10 μm. The data are presented as means ± SEMs. *n* = 30, 33 cells from three independent experiments. *p* values were calculated by Student’s *t*-test. ****p < 0.0001. The number of Rafeesomes containing endogenous LC3 was counted.

During phagophore expansion, ATG9A-embedded vesicles are recruited to the PAS and function in delivering membranes derived from diverse sources^45,46^. To determine whether ATG9A plays a role in the secretory ER-phagy, we transfected *ATG9A*^*–/–*^ HeLa cells with RTN4B-mCherry and found a pronounced decrease in the number of RTN4B-mCherry noncanonical autophagosomes, accompanied by an accumulation of RTN4B-mCherry vesicles (Fig. 8a). Moreover, GFP-ATG9A colocalized with mCherry-labeled RTN4B vesicles in *ATG5*^*–/–*^ HeLa cells, as well as RTN4B-mCherry noncanonical autophagosomes in HeLa cells (Fig. 8b). These results suggested a pivotal role of ATG9A in transporting RTN4 vesicles.

As expected, RAB22A-induced LC3-II levels and Rafeesome formation were abolished in cells with *ATG9A* depletion (Fig. 8c, d), consistent with the discovery that the formation of RTN4B noncanonical autophagosomes were blocked in *ATG9A*^*–/–*^ HeLa cells (Fig. 8a). In summary, we proposed that ATG9A participates in the RAB22A/TMEM33/RTN4 assembly-induced secretory ER-phagy pathway, promoting the formation of RTN4 noncanonical autophagosomes and subsequent secretion of ER components into R-EVs via Rafeesomes.

## Discussion

**Fig. 9:**
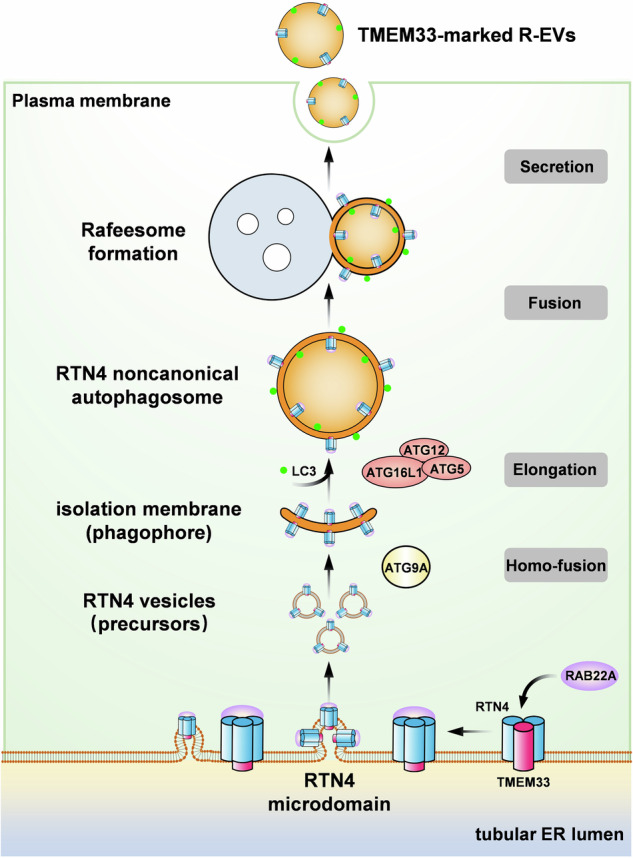
A proposed model deciphering a secretory ER-phagy pathway initiated by the assembly of RAB22A/TMEM33/RTN4. on the tubular ER membrane, RAB22A/TMEM33/RTN4 assembly promotes RTN4 oligomerization, which generates RTN4 cluster-enriched microdomains. ER remodeling resulted from RTN4 microdomains induces the bud scission of membrane-bound RTN4 vesicles. ATG9A transports RTN4 vesicles for homotypic fusion, leading to the formation of the early IM, which serves as phagophore of RTN4 noncanonical autophagosomes. Finally, the secretory ER-phagy-derived RTN4 noncanonical autophagosomes are secreted via Rafeesomes as R-EVs marked by TMEM33.

Previously, we demonstrated for the first time that ER proteins, such as STING and Calnexin, can be trafficked from the ER to Rafeesomes by riding RAB22A-mediated noncanonical autophagosomes and act as substrates of secretory autophagy^10^. In this study, as depicted in Fig. 9, we propose a secretory ER-phagy pathway where RAB22A/TMEM33/RTN4 assembly results in ER-remodeling. This event, in turn, drives the bud scission of RTN4 vesicles. Then, ATG9A transports these RTN4 vesicles to form the early IM, delivering membranes for RTN4-positive secretory autophagosomes, which are ultimately secreted by Rafeesomes as R-EVs marked by TMEM33. Along the secretory ER-phagy pathway, ER cargoes, even proteins lacking N-terminal signal peptides, can be incorporated into RTN4 noncanonical autophagosomes to be released into extracellular space.

Although autophagy is usually related to lysosomal degradation mechanism, accumulating evidence implicates autophagy in conventional or unconventional protein secretion (UPS) of multiple cytoplasmic cargoes. For instance, Deretic et al. found that starvation-induced autophagy cooperating with Golgi reassembly protein GRASP and RAB8A promotes IL-1β secretion, defining one type of unconventional secretion through autophagy machinery in mammalian cells^47^. Recently, Ge et al. identify a vesicle-mediated unconventional secretion pathway. RAB1s enhances TMED10 translocator activity, regulating cargo translocation into ERGIC, thereby driving a UcPS/THU pathway. During this process, TMED10 functions as a protein channel to regulate a subset of cargo lacking signal sequence release in autophagy-dependent manner^48,49^. Besides, Leidal et al. reported an autophagosome-independent secretory autophagy pathway termed LC3-dependent EV loading and secretion (LDELS) pathway, in which RNA-binding proteins (RBPs), like HNRNPK and SAFB, are loaded into MVBs via LC3-conjugation machinery for secretion^50^. A new UPS pathway that controls misfolding-associated protein secretion is delineated that misfolded proteins are encapsulated into ER-associated late endosomes to be secreted for protein quality control^51^. In addition, an EV composition reassessment experiment has proven that some cytoplasmic materials, such as DNA and histones, are released by amphisomes, hybrid structures formed by the fusion of autophagosomes with late endosomes during lysosome inhibition^52^. Our recent work has illustrated that RAB22A mediates a Rafeesome-R-EV route for cargoes secretion, like activated STING, through the trafficking of RAB22A-induced noncanonical autophagosomes^10^. In this study, we delineated that the secretory autophagosomes are formed by ER remodeling caused by the RTN4-clustering subdomains. Such secretory ER-phagy pathway multiplies the mechanisms for the secretion of ER components or cytosolic materials. Moreover, we identify TMEM33 as a biomarker for R-EVs, which may offer strategies for specifically separating R-EVs from other EV subpopulations to sort substrates for the secretory ER-phagy pathway.

In canonical autophagy, the formation of autophagosomes requires a finely-regulated conserved mechanism. Multiple ultrastructural analysis of the membrane source of canonical autophagy have unveiled that the phagophore structure emerges in close proximity to the ER. EM has shown that after starvation induction, DFCP1 forms a ring-like structure on the ER, which is termed omegasome because of its “Ω“ shape^53^. Omegasomes depend on LC3-II to mature into autophagic vacuoles. However, another model proposed that the ER might undergo deformation to generate an isolation membrane by in situ nucleation from the ER subdomain, appearing as a sandwich-like structure^54^. In addition, the contact sites between different membranes, such as the ER-Golgi (ERGIC), ER-mitochondria, ER-plasma membrane (PM) and ER-exit sites (ERES), have been reported to serve as cradles for the PAS due to abundant membrane and lipid resources^55–59^. We have revealed that RTN4 clustering facilitated by RAB22A/TMEM33/RTN4 assembly actively deformed the tubular ER membrane and led to the formation of RTN4-positive vesicles via bud scission, which is confirmed by super-resolution imaging (Fig. 6e, f). Interestingly, studies have shown that the IMs can be newly synthesized by the fusion of membrane structures. For instance, the formation of ATG16L1-positive phagophores requires the homotypic fusion of PM-derived ATG16L1 vesicles^59^. In addition, cis-Golgi-derived FIP200 vesicles heterogeneously fuse with endosome-derived ATG16L1-resident vesicles to form a hybrid pre-autophagosomal structure (HyPAS) that functions as the autophagosomal precursor membrane^60^. Our results demonstrated that overexpressed RTN4 induced substantial larger noncanonical autophagosomes (RTN4-positive, LC3-positive), but only inducing smaller vesicles (RTN4-positive, LC3-negative) in the absence of ATG12-ATG5-ATG16L1 complex (Fig. 6a–d). Likewise, deficiency in *ATG9A*-mediated trafficking of these vesicles was also suppressed the formation of noncanonical autophagosomes (Fig. 8a, d). Hence, we propose that, in RAB22A-mediated noncanonical autophagy, the synthesis of phagophore adopts a similar homo-typic fusion mechanism between these specific tubular ER-derived RTN4 vesicles.

In macro-ER-phagy, FAM134B is the most studied receptor that bridges ER fragments with phagophore^37,61,62^. Similar to RTN4, FAM134B contains an RHD domain and forms clusters that locally bend the membrane to fragment the ER^37,63^. Also, overexpression of FAM134B leads to the formation of punctae-like ER fragments decorated with LC3^37^. In addition, FAM134B locates mainly on the sheet ER to execute its function, but less on the ER tubule, as EM studies showed an accumulation of ribosomes-studded ER segments^37^. By contrast, our results support that RTN4-driven ER-phagy occurs primarily on the tubular ER with high curvature as the sheet ER marker Climp63 rarely enters into Rafeesomes (Fig. 1c). Particularly, FAM134B and RTN4 selectively deform the ER in different manners, although they both induce membrane curvature depending on RHD. It is possible that the interaction between FAM134B with LC3 via LIR generates an additional force on the membrane and easily fragments the ER^32^. Conversely, the ER membrane tends to bud in response to the only tension caused by RTN4 cluster-enriched microdomains, a process that does not require the LC3 binding, because small vesicles (RTN4-positive, LC3-negative) are still observed in *ATG5*- or *ATG16L1*-knock out cells (Fig. 6d). Indeed, it has been reported that FAM134B clusters also induce monolayer vesicles in ER subdomains with high curvature independent of autophagy machinery^42^. Notably, RTN3L, another ER-phagy receptor, has been identified as the only Reticulon protein that is capable of inducing ER fragments owing to the presence of six LIRs^38^. However, the “secretory ER-phagy” we described here differs fundamentally from traditional “ER-phagy”. Despite this distinction, both processes share a common substrate — the ER protein. Classical ER-phagy primarily involves the degradation of ER components, whereas secretory ER-phagy is characterized by the secretion of ER proteins outside the cell. Therefore, ER components may be endowed with completely different fates through diverse ER-phagy pathways either to be degraded or to be secreted. Multiple ER-phagy mechanisms may protect cells from various external or internal stimuli and provide alternative substitutions in case of perturbations of any of those pathways.

In summary, this study not only presents a new machinery in which the induction of noncanonical autophagy is contributed from the ER remodeling, but also discovers an RTN4-driven secretory ER-phagy pathway during Rafeesome biogenesis. We regard this secretory ER-phagy as an alternative strategy for cell in clearance of ER parts and regulating ER turnover. Furthermore, our findings reveal a close link between ER-phagy and secretory autophagy, providing insights into the secretion of ER cargoes.

## Materials and methods

### Antibodies and reagents

The following antibodies for western blotting and immunofluorescence analyses were used: anti-TMEM33 for WB (Abcam, ab242108); anti-TMEM33 for IF (Bethyl, A305-597AT); anti-RTN4 for WB (Proteintech, 10950-1-AP); anti-RTN4 for IF (Santa Cruz, sc-271878); anti-RAB22A (Sigma Aldrich, HPA066920); anti-LC3B for WB (Sigma Aldrich, L7543); anti-LC3B for IF (MBL International, PM036); anti-ATG9A (Abcam, ab108338); anti-ATG16L1 (8089 T), anti-ATG5 (12994) and anti-CD9 (13174) (Cell Signaling Technology); anti-Syntenin-1 (22399-1-AP); anti-ATL3 (16921-1-AP) and anti-Climp63 (16686-1-AP) (Proteintech); anti-CD63 (Beyotime, AF1471); anti-Calnexin (Santa Cruz, sc-46669); anti-TBC1D2B (Santa Cruz, sc-398906); anti-HA-tag (3724S), anti-FLAG-tag (14793S), anti-V5-tag (13202S) (Cell Signaling Technology); anti-GAPDH (Proteintech, 10494-1-AP); anti-β-Tubulin (Bioworld, AP0064); anti-FLOT2 (Cell Signaling Technology, 3436); anti-Alix (Proteintech, 12422-1-AP); anti-Vinculin (Santa Cruz, sc-73614); and secondary antibodies for IF conjugated with Alex Fluor-488 (A-11008), Alex Fluor-594 (A-11005), Alex Fluor-568 (A11036) and Alex Fluor-647 (A21236) were all from Invitrogen.

The following reagents were used in this study: DSS (Thermo Fisher, A39267); doxycycline (Selleck, S4163); poly-L-lysine (Beyotime, ST509); Hoechst (Invitrogen, 33342); ATP (MCE, HY-B0345A); anti-DYKDDDDK magnetic beads (Thermo Fisher, A36797); DAB (Sigma, D5637); 30% hydrogen peroxide (Sigma, 88597); anti-CD63 magnetic beads (Thermo Fisher, 10606D); anti-streptavidin Sepharose (Cytiva, 17511301); anti-DYKDDDDK agarose (Bimake, B23102); and anti-HA agarose (Bimake, B26201).

### Plasmid construction and truncation mutation

The following plasmids used in this study were constructed as previously described in our laboratory^10^: FLAG or GFP-tagged RAB22A^WT^ and the constitutively active mutant RAB22A^Q64L^. *RTN4* and *TMEM33* were amplified from HEK293T cDNA using high-fidelity PCR followed by cloning into a pSIN or pcDNA3.1 (+) vector with a tag (FLAG, HA, V5, APEX2, mCherry or GFP) utilizing the homologous recombination method. The GFP-ATG9A construct was a gift from Dr. Min Li (Sun Yat-sen University). RTN4 or TMEM33 truncation mutants were constructed following the instructions of the Fast Mutagenesis Kit. All the constructs used were verified by DNA sequencing.

### Cell culture and transfection

#### For cell culture

HEK293T and HeLa cell lines were obtained from ATCC. *ATG16L1*^*–/–*^ and *ATG5*^*–/–*^ HeLa cells were gifts from Dr. Feng Shao (Beijing Institute of Biological Science). *ATG9A*^*–/–*^ HeLa cell line was a gift from Dr. Min Li (Sun Yat-sen University). All the cell lines were cultured in Dulbecco’s modified Eagle’s medium (DMEM) supplemented with 10% FBS and 1% penicillin-streptomycin at 37 °C in an atmosphere of 5% CO_2_.

#### For transient transfection

Plasmids were transfected into cells with Lipofectamine 3000 (Life Technologies) according to the manufacturer’s instructions. After 4–6 h of transfection, the culture medium was replaced with fresh medium.

#### For lentivirus transfection

For virus packaging, when reaching 80% confluence, HEK293T cells were co-transfected with the packaging vectors psPAX2 and pMD2G together with the targeting plasmid or shRNA/sgRNA at the proper ratio using polyethylenimine (PEI, Polysciences). The culture medium was refreshed after 4–6 h. At 48–72 h post-transfection, the viral supernatants were collected and filtered through 0.45 μm filters (Millipore) to remove cell debris. To generate stable cell strains, the indicated cells were infected with the appropriate virus in the presence of 8 μg/mL polybrene (Sigma). After 24 h of infection, the pooled cells were selected in medium supplemented with 0.5 μg/mL puromycin (Macklin) to generate stable cell lines.

### Purification of total EVPs

EVP-deleted FBS was prepared by ultracentrifugation at 100,000× *g* for 24 h. Cells were cultured in complete medium in 150-mm culture dishes until they reached approximately 70% confluence. Then, the medium was discarded, and the cells were washed twice with PBS, followed by the addition of medium containing EVP-deleted FBS for 48 h. The culture medium was then harvested and subjected to sequential centrifugation (at 500× *g* for 10 min and 2000× *g* for 30 min) to fully remove cell debris. The supernatants were centrifuged twice at 100,000× *g* for 2 h to isolate total EVPs. The pellets were resuspended in proper sterile PBS, and then, BCA quantification analysis was performed following the manufacturer’s instructions to measure the EVP concentration. For long-term preservation, the EVPs were stored at –80 °C to avoid frequent freeze-thaw cycles.

### Immunoblotting and immunoprecipitation

For Western blotting analysis, cells were washed twice with ice-cold PBS and then lysed on ice in lysis buffer (50 mM Tris-HCl, pH 7.5, 150 mM NaCl, 1 mM EDTA, and 1% NP40) supplemented with protease inhibitor (Selleck). The lysates were centrifuged at 12,000× *g* for 20 min at 4 °C. For direct immunoblot analysis, the supernatants were resolved in 5× loading buffer and boiled for 5 min. For the immunoprecipitation assay, 40 μL of the supernatant was retained as the whole-cell lysate (WCL), and the remaining supernatant was incubated with the indicated agarose for 6 h at 4 °C. The agarose conjugated with the target protein was washed at least six times with lysis buffer followed by boiling in 5× loading buffer for 5 min. Appropriate amounts of WCL and agarose were loaded on and separated by 12% SDS-PAGE and then transferred to PVDF membranes (Millipore). The membranes were then blocked in 5% skim milk for 1 h at room temperature, followed by incubation with the indicated primary antibodies overnight at 4 °C. After being washed three times in PBS supplemented with 0.1% Tween-20, the membranes were incubated with HRP-conjugated secondary antibodies at room temperature for 1 h. The signal was detected by a MiniChemi Chemiluminescence imager (SAGECREATION, Beijing) after the membranes was treated with high-sig ECL substrate (Tanon).

### Immunofluorescence staining

Cells were treated as indicated. The medium was removed, and the cells were subsequently washed three times with PBS and fixed with 4% paraformaldehyde for 20 min at room temperature. After that, the cells were further permeabilized with 0.5% Triton X-100 for 15 min and blocked with goat serum for 1 h at room temperature. Then, the cells were incubated with the indicated primary antibodies overnight at 4 °C. Then, the cells were further incubated with secondary antibodies for 1 h at room temperature followed by incubation with Hoechst 33342 (Invitrogen) for 2 min. Images were obtained using a high-resolution laser confocal microscopy (Zeiss LSM880).

### SIM

SIM images were obtained using a Multi-SIM (Multimodality Structured Illumination Microscopy) imaging system (NanoInsights-Tech Co., Ltd.) equipped with a 100 × 1.49NA oil objective (Nikon CFI SR HP Apo). Images were taken using a single slice mode with 50 mW laser power and 50 ms exposure time, and were then reconstructed with the SIM Imaging Analyzer software (NanoInsights-Tech).

### 3D-STORM

In order to observe the ultrastructure of RTN4 vesicles, *ATG5*^*–/–*^ HeLa cells with RTN4B-FLAG transient expression were subjected to immunofluorescence staining. Then, the cells were mounted on coverslips treated with standard STORM imaging buffer (5% w/v glucose, 0.8 mg/mL glucose oxidase, 1 M cysteamine and 40 mg/mL catalase in Tris-HCl, pH 7.5). The reconstructed images were obtained in 3D-STORM mode performed on a homebuilt setup modified from Nikon Eclipse Ti-E inverted optical microscope (Nikon CFI Plan Apochromat l, 1003, numerical aperture = 1.45).

### Live-cell imaging

The formation of RTN4 puncta induced by RAB22A was recorded and observed using a Nikon Ti2 spinning disk microscope. Briefly, HeLa cells stably expressing tet-on-GFP-RAB22A^Q64L^ were cultured in a 35-mm glass-bottom dish followed by transfection with RTN4B-mCherry for 12 h the next day. Before imaging, the cells were treated with 50 ng/mL doxycycline, seeded in a chamber, and maintained at 37 °C with 5% CO_2_. Images were taken at 15-min intervals, and constantly collected for 12 h. Images were processed with NIS-Elements AR 5.30 software.

### DSS cross-linking

Oligomerization was detected by employing the DSS crosslinker, a membrane-permeable N-hydroxysuccinimide ester (NHS) that reacts efficiently with primary amino groups (-NH2) in pH 7 to 9 buffers to form stable amide bonds. Two milligrams of prepackaged DSS powder was dissolved in 216 μL of DMSO to prepare a 25 mM stock solution. Cells seeded on 12- or 6-well plates were washed twice with PBS (pH 8.0) to completely remove the remaining medium. Cells were treated with 500 μL of PBS (pH 8.0) containing 2 mM DSS, and placed on a horizontal shaker for 30 min at room temperature. Then, the DSS was discarded, and 500 μL of quenching solution (20 mM Tris-HCl, pH 7.5) was added for 15 min at room temperature to terminate the cross-linking reaction. Then, the cells were collected by scraping in 1× loading buffer and boiled for 10 min for subsequent immunoblot analysis.

### Suborganelle immunoprecipitation

To extract Rafeesomes or RTN4 noncanonical autophagosomes, the indicated cells plated in 15-cm dishes were washed twice with PBS and collected by scraping in 3 mL of cold KPBS buffer (136 mM KCl, 10 mM KH_2_PO_4_, pH 7.25 adjusted with KOH) on ice. Then, the cells were centrifuged at 2000× *g* for 3 min at 4 °C to precipitate the cell pellet. The pellet was resuspended in 500 μL of KPBS buffer containing protease inhibitor, and 40 μL of the suspension was kept as a whole-cell control. The remaining cells were transferred to a 2-mL dounce homogenizer (Sigma Aldrich) and gently homogenized with 40 strokes. The homogenates were further centrifuged at 5000× *g* for 5 min at 4 °C to remove cell debris and intact cells. The supernatant was transferred to a new tube containing anti-DYKDDDDK magnetic beads prewashed with KPBS buffer and incubated for 2 h at 4 °C on a rotator shaker. The beads were washed at least six times with KPBS buffer on a magnet, after which 40 μL of lysis buffer was added to beads, which were subsequently boiled in a metal bath in vibrating mode.

### In vitro LC3 lipidation

The in vitro LC3 lipidation assay was performed according to and slightly modified from published methods^64,65^. To obtain the cytoplasmic extract, wild-type HeLa cells grown in 15-cm dishes were washed with PBS and harvested in hypotonic buffer (20 mM HEPES-KOH, pH 7.2, 10 mM KCl, 3 mM MgCl_2_) containing protease inhibitor before being passed through a 25 G needle in no more than 4× cell pellet volume of hypotonic buffer. The cell lysate was centrifuged at 100,000× *g* for 2 h at 4 °C, and the supernatant was collected as the cytosolic extract. For membrane fraction preparation, *ATG5*^*–/–*^ HeLa cells seeded on 15-cm dishes were transiently transfected with vector, HA-TMEM33 or RTN4-FLAG respectively for 24 – 48 h, after which the cells were washed and homogenized in homogenization buffer (20 mM HEPES-KOH, pH 7.2, 400 mM sucrose, 0.5 mM EDTA) with a 2-mL dounce homogenizer. The homogenates were sequentially centrifuged (1000× *g* for 5 min, 5000× *g* for 10 min, 25,000× *g* for 30 min) to pellet the membrane fractions. For the in vitro LC3 lipidation reaction, cytosol (2 mg/mL final concentration), membrane fraction (0.5 mg/mL), ATP-regenerating system (1 mM ATP, 40 mM creatine phosphate, 0.2 mg/mL creatine phosphokinase), and GTP (0.15 mM) were mixed in a final volume of 30 μL, and the reaction was performed at 30 °C for 2 h followed by immunoblot analysis.

### In vitro fusion assay

The assay was performed based on a previously described method with some modifications^60,66^. In brief, *ATG5*^*–/–*^ HeLa cells transfected with GFP-RAB22A^Q64L^ and wild-type HeLa cells transfected with RTN4B-mCherry were homogenized in buffer (20 mM HEPES-KOH, pH 7.2, 250 mM sucrose, 1 mM EDTA) followed by centrifugation at 12,000× *g* for 15 min to prepare post-nuclear supernatants (PNSs). For the fusion reaction, both PNSs were mixed in the presence of an ATP regeneration system as described previously at 37 °C for 1 h by gently shaking. Then, the mixture was immobilized on a coverslip and observed using a digital whole-slide imaging scanner (KFBIO, KF-PRO-020) in fluorescent mode. The acquired images were analyzed using FIJI software, and at least 4000 puncta were quantified in each sample.

### Isolation of R-EVs and CD63^+^ exosomes

To distinctively purify the FLAG-labeled R-EVs and CD63-positive classical exosomes, total EVPs derived from the indicated cells were divided into two portions for incubation with anti-DYKDDDDK and anti-CD63 magnetic beads respectively in isolation buffer (PBS with 0.1% BSA, filtered through a 0.2-µm filter) overnight (18 – 22 h) at 4 °C. Then, the exosome-bound beads were washed at least six times with isolation buffer using a magnet. For immunoblot analysis, the beads were incubated with 40 µL of lysis buffer on ice for 15 min to lyse the exosomes followed by boiling for 5 min in 5× loading buffer. To obtain the pure R-EVs for immunofluorescence analysis, after washing with isolation buffer, the FLAG-tagged R-EV-bound beads were incubated with 200 µg/mL 3× FLAG peptide in elution buffer (10 mM Tris-HCl, pH 7.4, 150 mM NaCl) overnight at 4 °C to elute the R-EVs.

### APEX2-DAB staining for EM

GFP-RAB22A^Q64L^ HeLa cells transfected with APEX2-LC3, APEX2-TMEM33 or RTN4B-APEX2 or WT HeLa cells transfected with RTN4B-APEX2 were fixed with preheated 2.5% glutaraldehyde at room temperature for 15 min and quickly moved to ice for 30 min. Subsequent steps were performed on ice until resin infiltration. Cells were washed 3 × 5 min with cold 0.1 M PB buffer (0.02 M NaH_2_PO_4_, 0.08 M Na_2_HPO_4_, pH 7.2), followed by treatment with freshly diluted 0.5 mg/mL DAB solution combined with 0.03% (v/v) H_2_O_2_ for 30–60 min. To terminate the reaction, cells were washed 3 × 10 min with cold 0.1 M PB and post-fixed with 1% osmium tetroxide for 30 min. Then cells were washed with distilled water and dehydrated through a graded ethanol series (50%, 70%, 80%, 90%, 100% for 3 times; 10 min in each). Cells were transferred to room temperature and infiltrated with Pon 812 Resin by incubating the samples in a diluted series of ethanol-Pon 812 at a 1:1, 1:2 and 1:3 ratios for 2 h each, and then overnight in pure resin in an oven at 60 °C for 48 h. Samples were sliced into 70-nm-thick sections by ultramicrotome (Leica EM UC7). Samples were observed with the HT-7800 120 kv transmission electron microscope.

### Statistical analysis

Statistical analysis was performed using GraphPad Prism 8.3.0. All data are presented as means ± SEMs. Significant differences between two groups were evaluated by Student’s *t*-test. *p* < 0.05 was considered significant. *p < 0.05, **p < 0.01, ***p < 0.001, ****p < 0.0001.

## Supplementary information

Supplementary Table S1

Supplementary Table S2

Supplementary Figures

Supplementary Video S1
