# Supplementary material for: The assembly of RAB22A/TMEM33/RTN4 initiates a secretory ER-phagy pathway
Source: Cell Discov. 2025 Apr 29;11:41. doi: 10.1038/s41421-025-00792-2 (PMC12041605; doi:10.1038/s41421-025-00792-2)
Supplement: Supplementary file 3 — Supplementary Figures [file 41421_2025_792_MOESM3_ESM.pdf]

Supplementary information

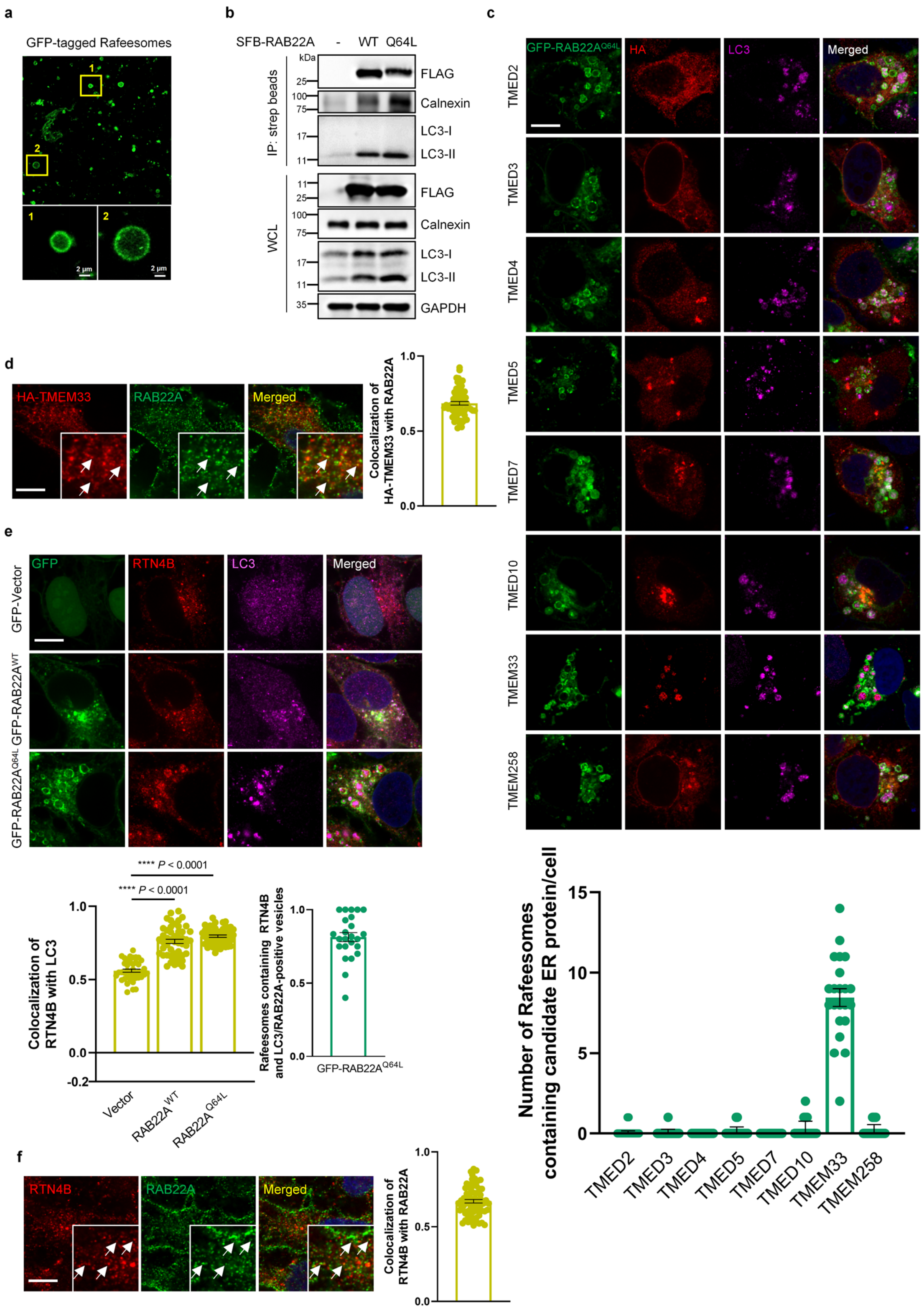

g

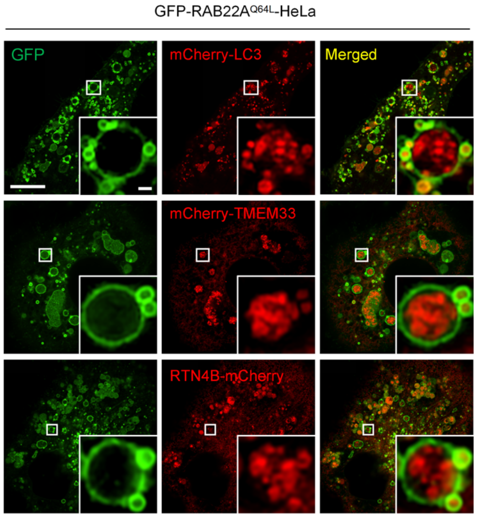

Supplementary Fig. S1. The ER membrane proteins TMEM33 and RTN4B are both enriched in Rafeesomes.

a Intact purified GFP-tagged Rafeesomes (1~5 μm in diameter) extracted from HeLa cells stably expressing GFP-RAB22A^{Q64L} were observed via fluorescence microscopy. Scale bar, 2 μm. **b** The purified SFB-tagged Rafeesomes were verified through Western blotting using the indicated antibodies. **c** HeLa cells stably expressing GFP-RAB22A^{Q64L} were individually transfected with partial screened HA-tagged ER transmembrane proteins identified from MS results. Immunofluorescence was performed to detect the colocalization of GFP-RAB22A^{Q64L} (green), HA (red) and endogenous LC3 (magenta). Scale bar, 10 μm. n ≥ 20 cells from three independent experiments. **d** HeLa cells were transfected with HA-TMEM33, and the colocalization of endogenous RAB22A (green) with HA (red) was detected by. Scale bar, 10 μm. Quantification of TMEM33 colocalization with RAB22A was presented as Pearson's correlation coefficient (*r*). n = 63 cells from three independent experiments. **e** The colocalization of endogenous RTN4B (red) and LC3 (magenta) with GFP-RAB22A (green) was observed in HeLa cells stably expressing GFP-Vector、GFP-RAB22A^{WT} or GFP-RAB22A^{Q64L}. Scale bar, 10 μm. Quantification of RTN4B colocalization with LC3 was presented as Pearson's correlation coefficient (*r*). The data are presented as means ± SEMs. n = 41, 49, 50 cells

from three independent experiments. *P* values were calculated by Student's *t* test. ****$P < 0.0001$. The ratio of Rafeesomes containing endogenous RTN4B and LC3 was quantified and calculated relative to total RAB22A-positive vesicles. **f** The colocalization of endogenous RTN4B (red) and RAB22A (green) was detected in HeLa cells. Scale bar, 10 μm. Quantification of RTN4B colocalization with RAB22A was presented as Pearson's correlation coefficient (*r*). n = 71 cells from three independent experiments. **g** HeLa cells stably expressing GFP-RAB22A^{Q64L} were transiently transfected with mCherry-LC3, mCherry-TMEM33 and RTN4B-mCherry respectively, then the ILV structures within Rafeesomes were imaged by a super-resolution confocal microscopy in live cells. Scale bar, 10 μm. Inset, 0.5 μm.

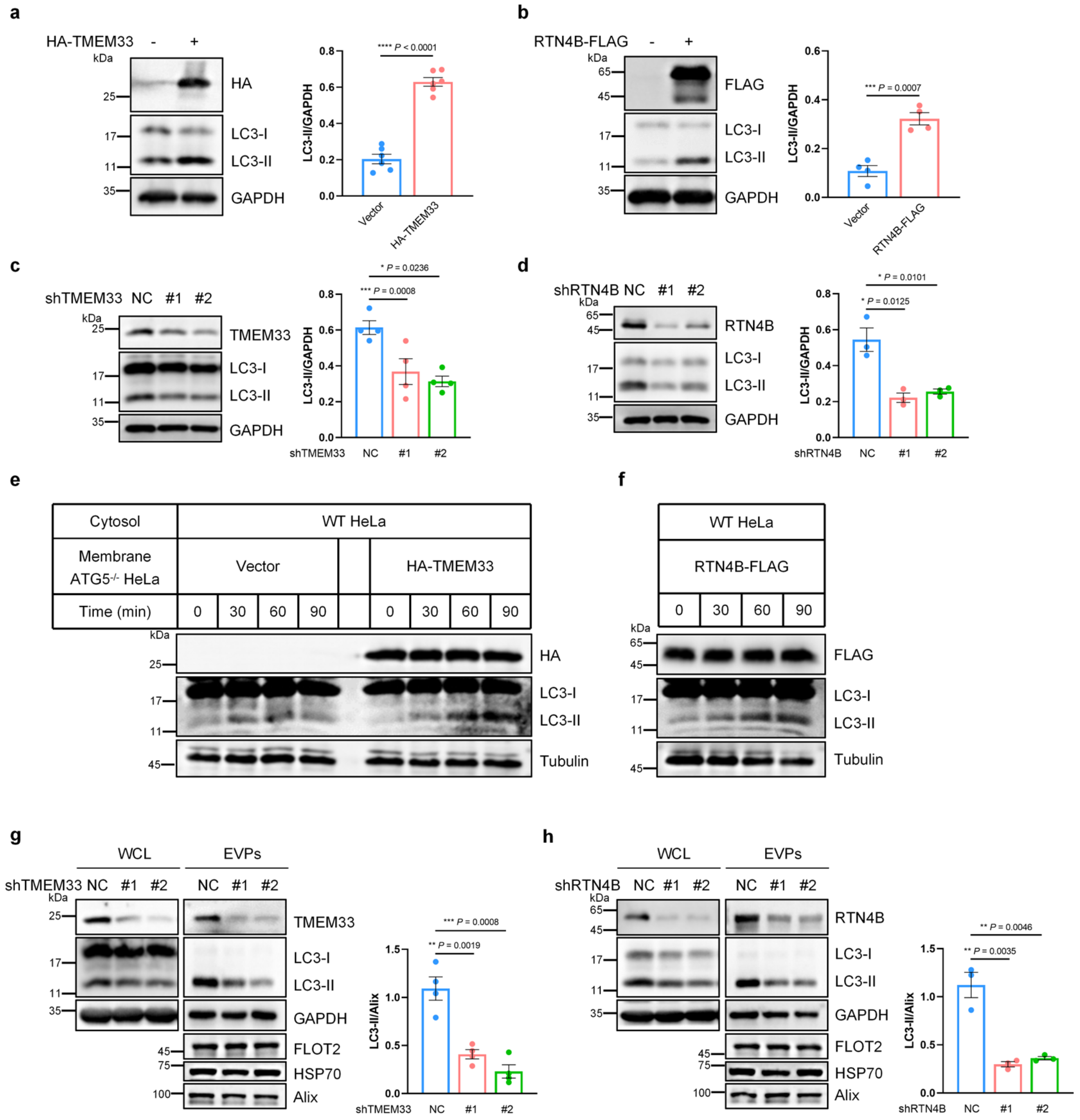

Supplementary Fig. S2. TMEM33 and RTN4 both induce autophagy.

a, b The LC3-II levels in HeLa cells transiently expressing HA-TMEM33 (a) or RTN4B-FLAG (b) were analyzed by Western blotting. The data are presented as means ± SEMs. n ≥ 4. *P* values were calculated by Student's *t* test. ****P* < 0.001, *****P* < 0.0001. **c, d** The LC3-II levels in HeLa cells with the knockdown of TMEM33 (c) or RTN4B (d) were analyzed by Western blotting. The data are presented as means ± SEMs. n ≥ 3. *P* values were calculated by Student's *t* test. **P* < 0.05, ****P* < 0.001. **e, f** The cytosol of WT HeLa cells and the membrane fractions of *ATG5*$^{-/-}$ HeLa cells overexpressing HA-TMEM33 (e) or RTN4B-FLAG (f) were incubated for 0-90 min at 30 °C. Then, the mixtures were

analyzed via Western blotting, and the relevant lipidated LC3 levels were measured. **g, h** EVPs collected from HeLa cells with the knockdown of TMEM33 (g) or RTN4B (h) were subjected to Western blotting, and the secretion of LC3-II was detected. The data are presented as means ± SEMs. n ≥ 3. *P* values were calculated by Student's *t* test. $^{**}P < 0.01$, $^{***}P < 0.001$.

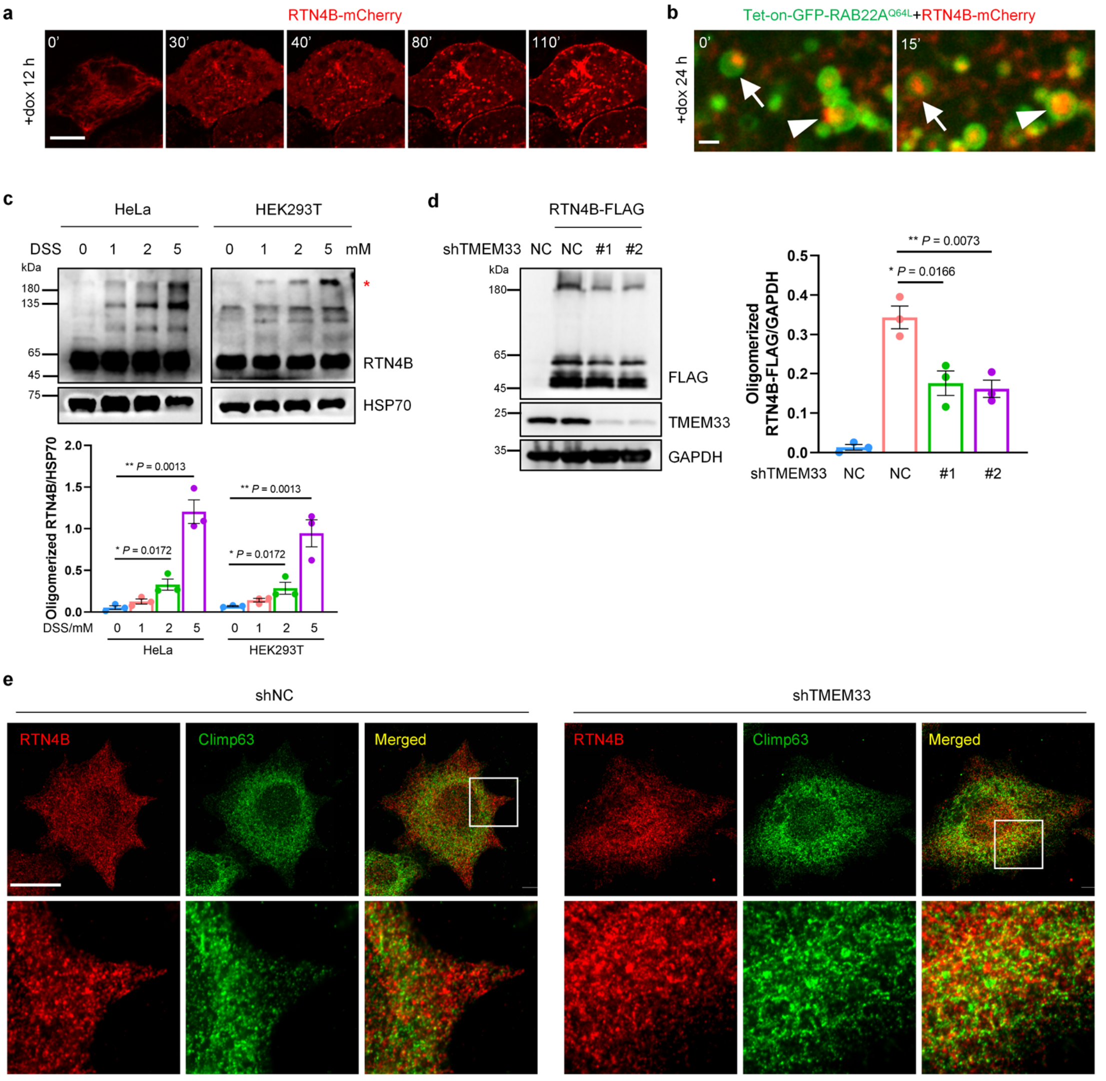

Supplementary Fig. S3. Both RAB22A and TMEM33 promote RTN4 oligomerization to form RTN4 puncta.

a Tet-on-GFP-RAB22A^{Q64L} stable HeLa cells were transiently transfected with RTN4B-mCherry for 24 h, followed by the addition of 50 ng/mL dox for 12 h. Then time-lapse imaging was performed with a Nikon Ti2 spinning disk living cell microscope. Increased RTN4B-mCherry puncta formation was observed. Scale bar, 10 μm. **b** Living cell imaging showed the entry of RTN4B-mCherry puncta into dox-inducible Rafeesomes. Scale bar, 1 μm. **c** Both HeLa and HEK293T cells were treated with the indicated concentrations of DSS, and endogenous RTN4B oligomerization was analyzed via Western blotting. The data are presented as means ± SEMs. n = 3. *P* values were calculated by Student's *t* test. **P* < 0.05, ***P* < 0.01. **d** HeLa cells with TMEM33-knockdown

were transiently transfected with RTN4B-FLAG, and RTN4B-FLAG oligomerization was assessed by Western blotting. The data are presented as means ± SEMs. n = 3. *P* values were calculated by Student's *t* test. **P* < 0.05, ***P* < 0.01. **e** Immunofluorescence of endogenous RTN4B and Climp63 in HeLa cells with or without TMEM33 downregulation. Scale bar, 10 μm.

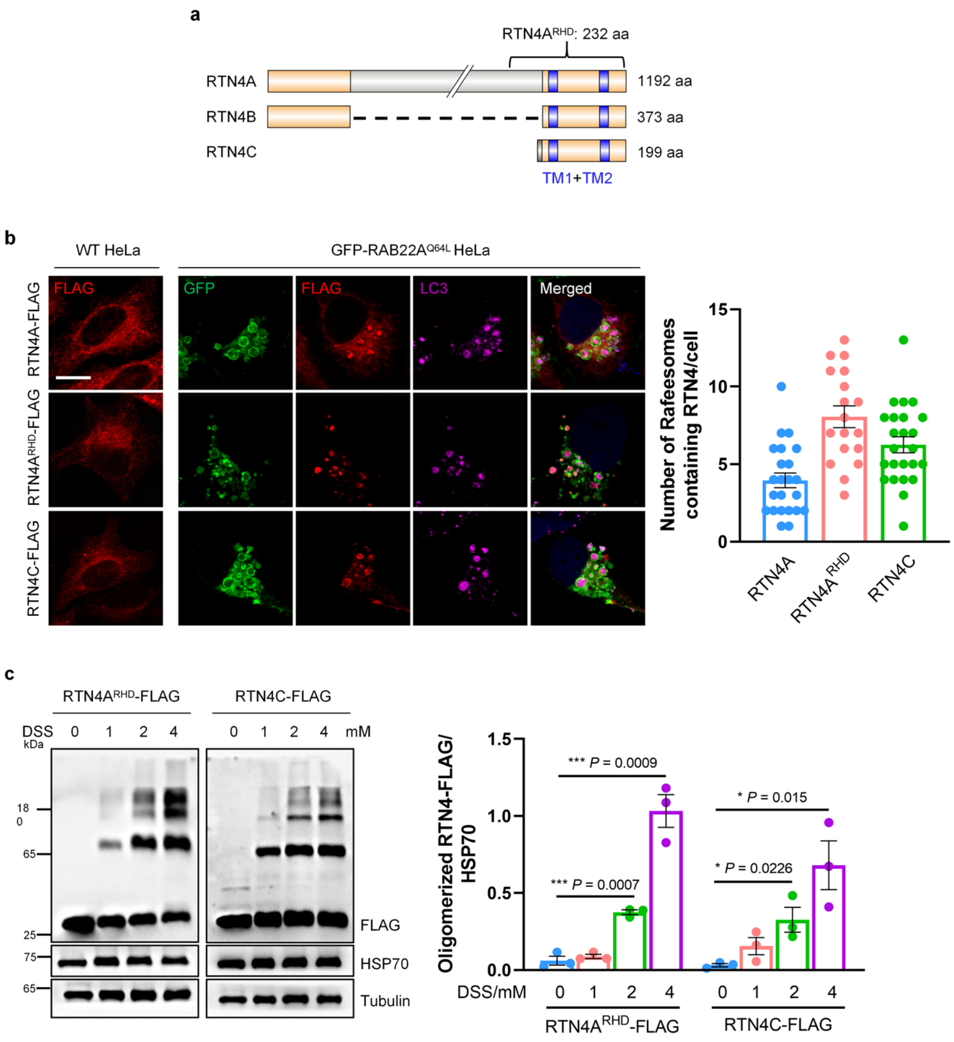

Supplementary Fig. S4. The Reticulon4 family may participate in the formation of RAB22A-mediated noncanonical autophagosomes.

a Diagram showing the RHD in the Reticulon4 family structure. **b** The entry of FLAG-tagged RTN4A, RTN4A^{RHD} or RTN4C into Rafeesomes was observed in HeLa cells stably expressing GFP-RAB22A^{Q64L}. Scale bar, 10 μm. n = 23, 18, 24 cells from three independent experiments. The number of Rafeesomes containing different RTN4 isoforms was counted. **c** HEK293T cells were transiently transfected with RTN4A^{RHD}-FLAG or RTN4C-FLAG followed by treatment with the indicated concentrations of DSS, and the oligomeric patterns of RTN4A^{RHD}-FLAG and RTN4C-FLAG were analyzed via Western blotting. The data are presented as means ± SEMs. n = 3. *P* values were calculated by Student's *t* test. *$P < 0.05$, ***$P < 0.001$.

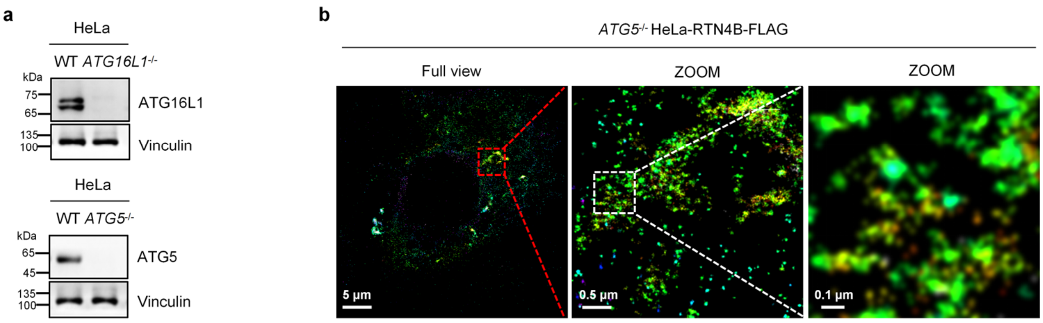

Supplementary Fig. S5. RTN4-enriched microdomains bud off from the ER membrane to form RTN4 vesicles.

a Verification of Knockout efficiency of *ATG16L1*$^{-/-}$ or *ATG5*$^{-/-}$ HeLa cells. **b** 3D-STORM analysis of small RTN4B-FLAG vesicles in *ATG5*$^{-/-}$ HeLa cells transiently transfected with RTN4B-FLAG.

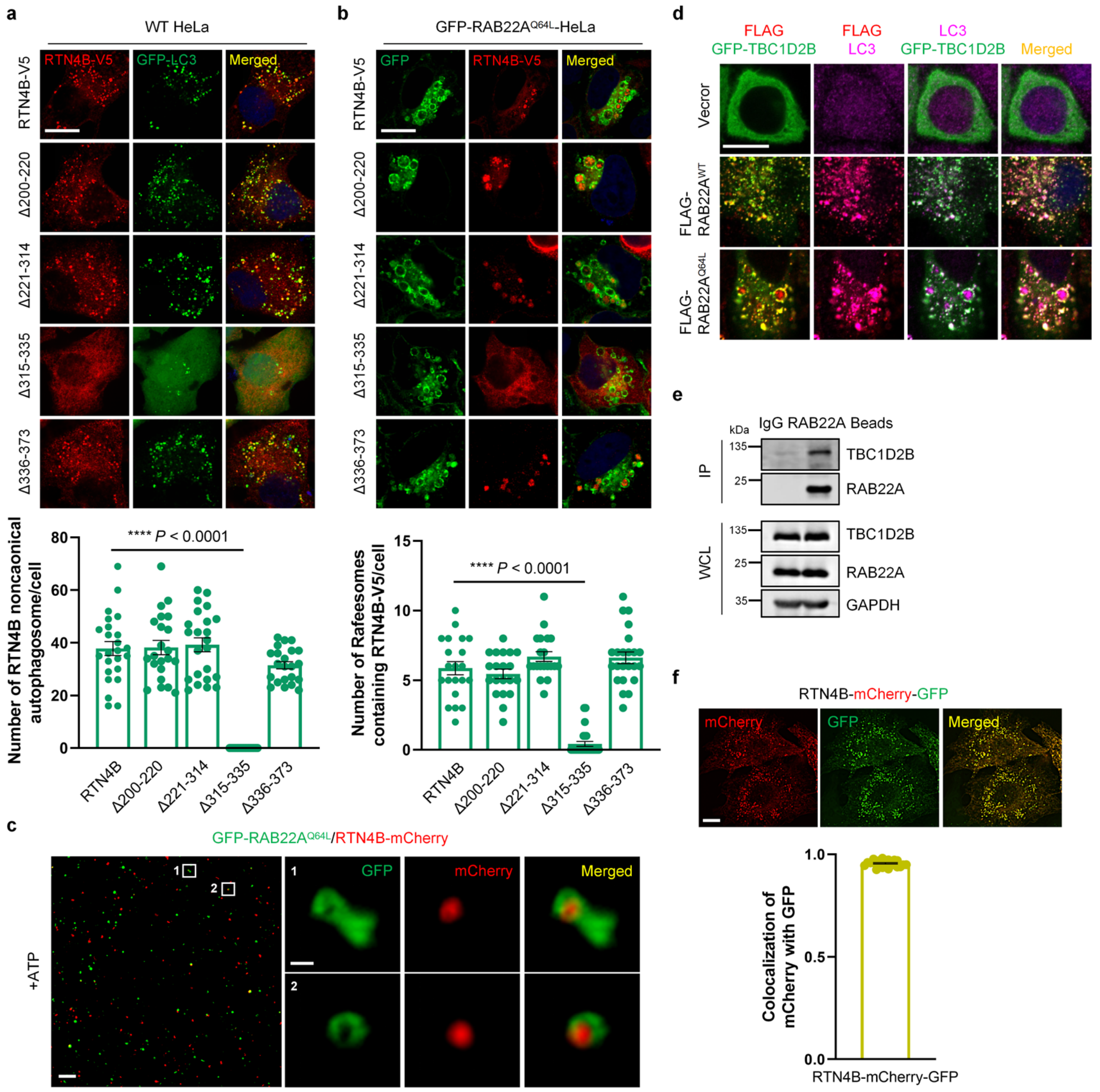

Supplementary Fig. S6. RTN4 noncanonical autophagosomes are protected from lysosomal degradation.

a HeLa cells were co-transfected with GFP-LC3 and V5-tagged RTN4B-truncation mutants, after which the formation of RTN4B-V5 noncanonical autophagosomes was assessed. Scale bar, 10 μm. The data are presented as means ± SEMs. n ≥ 22 cells from three independent experiments. *P* values were calculated by Student's *t* test. ****$P < 0.0001$. **b** HeLa cells stably expressing GFP-RAB22A^{Q64L} were transfected with V5-tagged RTN4B-truncation mutants, after which the formation of Rafeesomes was assessed. Scale bar, 10 μm. The data are presented as means ± SEMs. n ≥ 20 cells from

three independent experiments. *P* values were calculated by Student's *t* test. $^{****}P < 0.0001$. The number of Rafeesomes containing RTN4B-V5 noncanonical autophagosomes was counted. **c** Cytosols extracted from *ATG5*$^{-/-}$ HeLa cells stably expressing GFP-RAB22A^{Q64L} and HeLa cells transfected with RTN4B-mCherry were mixed in the presence of ATP for *in vitro* fusion assay. The fusion events were detected by a super-resolution confocal microscopy. Scale bar, 5 μm. Inset, 1 μm. **d** The colocalization of GFP-TBC1D2B (green) and endogenous LC3 (magenta) with FLAG-RAB22A (red) was observed in HeLa cells stably expressing Vector、FLAG-RAB22A^{WT} or FLAG-RAB22A^{Q64L}. Scale bar, 10 μm. **e** The cell lysates from HeLa cells were incubated with anti-RAB22A-conjugated agarose overnight, then the protein-bound beads was subjected to Western blotting to detect the interaction between RAB22A and TBC1D2B. **f** HeLa cells were transfected with RTN4B-mCherry-GFP. Quantification of mCherry colocalization with GFP was presented as Pearson's correlation coefficient (*r*). n = 24 cells from three independent experiments.

Supplementary Video S1. Time-lapse of image of RTN4B-mCherry in living *ATG5*$^{-/-}$ HeLa cells transiently expressing RTN4B-mCherry.
